# Fabrication of 3D-Printed Scaffolds with Multiscale Porosity

**DOI:** 10.1021/acsomega.3c09035

**Published:** 2024-06-28

**Authors:** Rafał Podgórski, Michał Wojasiński, Artur Małolepszy, Jakub Jaroszewicz, Tomasz Ciach

**Affiliations:** † Faculty of Chemical and Process Engineering, Warsaw University of Technology, Waryńskiego 1, 00-645 Warsaw, Poland; ‡ Faculty of Materials Science and Engineering, Warsaw University of Technology, Wołoska 141, 02-507 Warsaw, Poland; § Centre for Advanced Materials and Technologies, CEZAMAT, Poleczki 19, 02-822 Warsaw, Poland

## Abstract

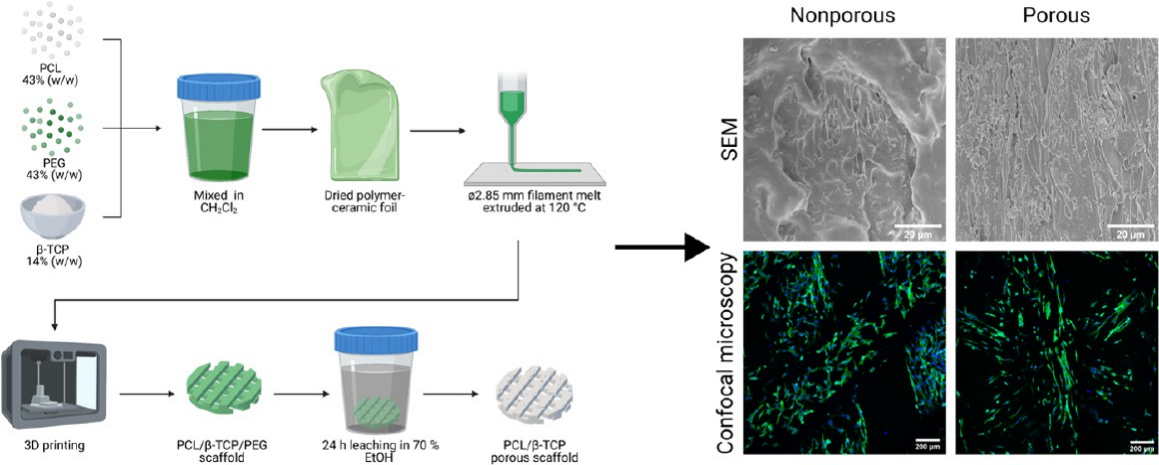

3D printing is a promising technique for producing bone implants, but there is still a need to adjust efficiency, facilitate production, and improve biocompatibility. Porous materials have a proven positive effect on the regeneration of bone tissue, but their production is associated with numerous limitations. In this work, we described a simple method of producing polymer or polymer-ceramic filaments for 3D-printing scaffolds by adding micrometer-scale porous structures on scaffold surfaces. Scaffolds included polycaprolactone (PCL) as the primary polymer, β-tricalcium phosphate (β-TCP) as the ceramic filler, and poly(ethylene glycol) (PEG) as a porogen. The pressurized filament extrusion gave flexible filaments composed of PCL, β-TCP, and PEG, which are ready to use in fused filament fabrication (FFF) 3D printers. Washing of 3D-printed scaffolds in ethanol solution removed PEG and revealed a microporous structure and ceramic particles on the scaffold’s surfaces. Furthermore, 3D-printed materials exhibit good printing precision, no cytotoxic properties, and highly impact MG63 cell alignment. Although combining PCL, PEG, and β-TCP is quite popular, the presented method allows the production of porous scaffolds with a well-organized structure without advanced equipment, and the produced filaments can be used to 3D print scaffolds on a simple commercially available 3D printer.

## 1 Introduction

Bone is the second most transplanted human tissue after blood transfusions,^1^ and the global market for orthopedic implants is estimated at $33.5 billion.^2^ The need for new materials development and the production of bone implants results from severe limitations of the still most popular method of bone grafting from the same patients or bone harvested from donors.^3,4^ However, artificial bone implants must be characterized by appropriate biocompatibility with native bone tissue, have proper mechanical properties, and enable the regeneration of the patient’s original tissue.^5^ One of the critical parameters in the design and manufacture of bone implants is their porous structure. High porosity with interconnected pores is necessary for bone cell migration, blood vessel ingrowth, and diffusion of nutrients, leading to an integration of the implant with surrounding tissue.^6,7^ There is no consensus on the implant pore size for the fastest bone regeneration, but the best osteogenic and osteoconductive properties, with sufficient mechanical strength, were typically obtained for the pore size range from 100 to 800 μm, as reported in the literature.^5,7−9^ Pores with smaller sizes are also important for properly integrating the implant into the bone tissue. Osteoblasts attach and grow better on surfaces with higher roughness,^10,11^ and surface pores, ridges, or grooves with size below 10 μm stimulate bone protein absorption, ion exchange, and bone-like apatite formation.^9,12,13^ Combining pores of different dimensions is an important issue called multiscale porosity because it gives a better therapeutic effect than using pores of one size.^7,14^

There are several ways to produce porous materials for bone implantation.^9^ Methods using rapid prototyping techniques, such as additive manufacturing technologies, are growing in importance because using 3D-printing techniques allows obtaining of porous scaffolds with a designed and complex geometry, which was difficult to achieve with traditional methods.^15^ This approach allows the scaffold to be shaped to match the cavities of individual patients, and the ability to plan the layout of the beams allows the density and porosity to be controlled to obtain the best approximation to the parameters of the original tissue.^15^ The most widely studied technique for 3D-printing bone implants is the fused filament fabrication (FFF) technique, where a molten thermoplastic material is extruded by nozzle.^16,17^ Another popular technique for 3D-printing bone implants is the powder bed fusion (PBF) technique, particularly in selective laser sintering (SLS), which is mainly used to acquire porous metal bone implants. The 3D-printing method, regardless of its type, allows receiving implants from various materials, such as biodegradable polymers (polylactic acid—PLA, polycaprolactone—PCL^18,19^), ceramic (β-tricalcium phosphate—β-TCP,^20^ hydroxyapatite—HA^19,21^), or metals (titanium alloys^22^). The disadvantage of this method is that the minimum pore size is limited by the accuracy of the selected 3D-printing method—for the FFF technique, 3D-printing *z*-axis resolution is 50–200 μm,^23^ and for the SLS technique, the *z*-axis resolution is 30–80 μm.^23,24^

To create pores with smaller sizes, a better choice is to use classical processing methods where the precision of the 3D printer does not limit the pore size but by the size of the used particles or controlled gas bubble formation. The first such method is solvent casting/particulate leaching, which is based on mixing the polymer solution with particles of substances soluble in water, such as NaCl of sucrose. After solvent evaporation, the obtained polymer matrix with crystal particles can be immersed in water to remove salt or sugar, achieving a porous structure.^25−27^ These methods allow the production of highly porous scaffolds, but pores are mostly unconnected and their size strongly depends on the size of used particles. Leaching is limited to thin materials, mostly because of the low water penetration of the polymer matrix, and the shape of scaffolds strongly depends on the mold geometry used. Yet another method, solvent-induced phase separation, allows for obtaining highly porous structures in polymer materials by using a difference in polymer solubility in different organic solvents and different solvents’ boiling point temperatures.^28^ Gas-foaming is another process where polymers are subjected to high pressure with gas-foaming agents such as CO_2_, supercritical CO_2_, nitrogen, or water.^9,29^ A sudden change in pressure and temperature results in nucleation and the growth of gas bubbles with a size range of 10–500 μm. The gas-foaming process is free of organic solvents, but the obtained materials have unconnected pores and nonporous external surfaces.^25^

The methods mentioned above have significant limitations, like using expensive equipment, resulting in unconnected pores, or limited geometry of produced materials, which impede implementing porous materials in large-scale treatment of bone defects. The solution to this problem may come from applying poly(ethylene glycol) as a porogen. Poly(ethylene glycol) (PEG) is a synthetic polymer, hydrophilic polyether,^25^ highly soluble in water and the broad spectrum of organic solvents like, e.g., ethanol, dichloromethane, benzene, and tetrahydrofuran.^30−32^ PEG is approved by the Food and Drug Administration for medical use^33^ and has wide use in commercial applications, especially in medicine,^34,35^ biotechnology,^36^ cosmetology,^37^ and the food industry.^38^ PEG properties allow easy mixing with other polymers in organic solvent solutions, and water solubility allows for subsequent removal of the PEG without disturbing the other components of the composite that are insoluble in water. Such properties of PEG were used by Bhaskar et al.,^39^ who used poly(ethylene glycol) as an additional porogen in the sugar leaching process of polylactic acid porous scaffold production. Similar usage was presented by Scaffaro et al., who used the addition of PEG to make connections between larger pores in the salt leaching method.^40^ An analogous way of using PEG in the salt leaching process was also presented by Scaffaro et al. in scaffolds made of PCL.^41^ An example of PEG’s use for 3D printing is the PLA scaffolds with the addition of PEG obtained by Serra et al.^42^ and Salehi et al.^43^ Despite the visible effect of the PEG addition on the faster degradation of the scaffolds, the obtained materials showed no signs of pores with sizes below 10 μm. So far, pore sizes resulting from the geometry of the scaffold model have been reduced.^42^

Researchers have shown that PEG can be used as a porogen in scaffolds for bone tissue engineering, but the appearance of the porous structure requires additional treatment. That is why we used PEG to obtain porous bone implants with micrometer-sized surface pores by FFF 3D printing followed by PEG leaching. Such materials could significantly reduce costs and accelerate the production of porous scaffolds for bone implants. Thus, we hypothesize that the proposed composition of PCL and PEG as a composite for 3D-printing bone implants should result in proper scaffolds. This is due to a similar range of PCL and PEG melting point temperatures, for each polymer’s molecular weight range. To investigate this hypothesis, we decided to use our method of producing polymer and polymer-ceramic filaments^44,45^ to obtain filaments from PCL, or composite filaments of PCL with the addition of β-TCP, and as porogen, we used two types of PEGs, differing in molecular weight. The use of PCL comes from the polymer’s proven biocompatibility and mechanical properties for producing bone implants.^19,46^ At the same time, PCL’s low melting point (56–65 °C) allows mixing with materials sensitive to higher temperatures, such as PEG.^47^ We also chose β-TCP as the ceramic material because it is one of the most widely tested sources of calcium and phosphorus, essential components of the mineral part of bone.^48^ Appropriately processed PCL and β-TCP composite into the bone tissue scaffolds have proven proper morphology and mechanical properties,^49^ as well as osteoinductive and osteoconductive properties.^26,50^ The filaments described above were substrates for a commercial FFF 3D printer to examine whether the 3D-printed scaffolds showed designed physical and biological properties to be candidates for bone tissue implants.

## 2 Materials and Methods

### 2.1 Materials

Polycaprolactone (PCL, *M*_n_ = 80,000 g·mol^–1^, Sigma-Aldrich), poly(ethylene glycol) (PEG4, *M*_n_ = 4000 g·mol^–1^, Clariant), poly(ethylene glycol) (PEG20, *M*_n_ = 20,000 g·mol^–1^, Merck), dichloromethane (DCM, Chempur), β-tricalcium phosphate (β-TCP, Sigma-Aldrich), Dulbecco’s phosphate-buffered saline (DPBS, Thermo Fisher), Dulbecco’s modified Eagle’s medium (DMEM, Thermo Fisher Scientific), 10,000 U·mL^–1^ penicillin and 10,000 μg·mL^–1^ streptomycin solution (Pen/Strep, Thermo Fisher Scientific), fetal bovine serum (FBS, Thermo Fisher Scientific), Trypsin-EDTA, 0.25% solution with phenol red (Thermo Fisher Scientific), The Cell Proliferation Kit II [XTT] (XTT, Roche), Alizarin-Red S (ARS, Sigma-Aldrich), cetylpyridinium chloride monohydrate (Sigma-Aldrich), 4,6-diamidino-2-phenylindole (DAPI, Thermo Fisher Scientific), Triton X-100 (Merck), bovine albumin fraction V (BSA, Roth), Alexa Fluor 488 Phalloidin (Thermo Fisher Scientific), ethanol 96% (Chempur), paraformaldehyde (PFA, Sigma-Aldrich), L929 cell line (Merck), MG63 cell line (Merck), QuantiPro BCA Assay Kit (Merck), 4-nitrophenyl phosphate disodium salt hexahydrate (Merck), RIPA Lysis and Extraction Buffer (Thermo Fischer Scientific), magnesium chloride (Chempur), sodium hydroxide (Chempur).

### 2.2 Preparation of PCL Foils with Different Concentrations of PEG

PCL and PEG foils were prepared by dissolving the polymers in DCM and mixing them overnight with a magnetic stirrer. The concentrations of PCL in the solutions and the amounts of PCL, PEG, and DCM used are listed in Table 1. The prepared solutions were dispensed using an Elcometer 3700 device, set to 0.1 mm, on a glass plate. The poured materials were dried overnight at 40 °C, and the dried foils were cut into disks with a diameter of 14 mm.

**Table 1:** Composition of PCL and PEG Mixtures for Foil Preparation

sample name	PCL [g]	PEG4 [g]	PEG20 [g]	DCM [mL]
PCL	3.00			30
PCL-PEG4-10%	3.00	0.33		33
PCL-PEG4-20%	3.00	0.75		37.5
PCL-PEG4-30%	3.00	1.29		43
PCL-PEG4-40%	3.00	2.00		50
PCL-PEG4-50%	1.50	1.50		30
PCL-PEG20-10%	3.00		0.33	33
PCL-PEG20-20%	3.00		0.75	37.5
PCL-PEG20-30%	3.00		1.29	43
PCL-PEG20-40%	3.00		2.00	50
PCL-PEG20-50%	1.50		1.50	30

### 2.3 Preparation of Polymer and Polymer-Ceramic Filaments

For each variant, 20 g of PCL and 20 g of PEG4 or PEG20 were dissolved in 60 mL of DCM and mixed overnight. Solutions of polymers with 25% (w/w based on the PCL and β-TCP weight) addition of β-TCP (approximately 1 to 10 μm in particle size^51^) were obtained by adding an adequate amount of β-TCP and 20 mL of DCM and mixing for 6 h. In all variants with β-TCP, the mass of β-TCP was 25% of the total mass of the PCL + β-TCP mix, assuming complete leaching out of PEG. All of the variants of the polymer and polymer-ceramic concentrations in the final composites are presented in Table 2. Solutions of the plain polymer and polymer with β-TCP were poured onto a flat glass bed and dried at 40 °C for 24 h. The obtained polymer and polymer-ceramic foils were cut into 5 × 1 cm^2^ strips and melted at 120 °C for 15 min in a stainless steel container with a pressure filament extruder. Our team developed the device and protocols used, and all details of the construction are presented in previous publications.^44,45^ The melted polymer was extruded through a 2.85 mm nozzle by using an air pressure of 4 bar. The obtained filaments were collected on a flat steel bar as 1 m long segments. Once the material was depleted, the device was disassembled and a new stainless steel container and nozzle were installed to repeat the process with different material variants.

**Table 2:** Composition of Polymer and Polymer-Ceramic Materials for Filament Preparation

sample name	PCL (% w/w)	β-TCP (% w/w)	PEG4 (% w/w)	PEG20 (% w/w)
PCL	100	0	0	0
PCL-BTCP	75	25	0	0
PCL-PEG4	50	0	50	0
PCL-BTCP-PEG4	43	14	43	0
PCL-PEG20	50	0	0	50
PCL-BTCP-PEG20	43	14	0	43

### 2.4 Scaffolds 3D Printing

Two distinct scaffold designs were developed using AutoCAD 2016 (Autodesk) for separate research objectives. The scaffolds for cell culturing, Alizarin-Red S staining, and surface analysis were created as 14 mm diameter discs with a height of 1.2 mm and comprised three beams tilted at a 60° angle to one another (Figure 3A). Each beam was 0.4 mm tall, 0.3 mm wide, and the space between them was 0.8 mm. The scaffolds for the mechanical test were designed as 12 mm diameter cylinders with a height of 12 mm and were constructed from 30 layers of beams that were tilted at a 60° angle relative to the previous layer (Figure 3C). Each beam was 0.4 mm tall, 0.3 mm wide, and the gap between them was 0.6 mm. Both designs were derived from common geometries used for the 3D printing of bone implants.^52^ The designed models were exported as STL files, and Voxelizer 2 software was used to generate GCODE files for 3D printing. The scaffolds were 3D-printed in a ZMorph VX commercial 3D printer (ZMorph) using filaments as described in Section 2.3 (Table 2) at a temperature of 160 °C. Photographs of the 3D-printed scaffolds were taken by using an iPhone 13 (Apple).

### 2.5 PEG Leaching Procedure

PCL-PEG foils (see Section 2.2) were washed by shaking (250 rpm) in 2 mL of ethanol solution in water (70% v/v) for 24 h in a DTS-4 shaker (ELMI). Next, the foils were rinsed twice in a fresh ethanol solution and dried at 40 °C for 24 h. Scaffolds for cell culturing, Alizarin-Red S staining, and surface analysis (see Section 2.4) were washed by shaking (250 rpm) in 2 mL of ethanol solution in water (70% v/v) for 24 h in a DTS-4 shaker. Next, the scaffolds were rinsed twice in a fresh ethanol solution and dried at 40 °C for 24 h. Scaffolds for mechanical testing (see Section 2.4) were washed by shaking (250 rpm) in 50 mL of ethanol solution in water (70% v/v) for 24 h in a DTS-4 shaker. Next, the scaffolds were rinsed twice in a fresh ethanol solution and dried at 40 °C for 24 h. All foils and scaffolds were weighed before and after leaching to calculate the weight loss. The porosity of the scaffolds before and after leaching was calculated using eq 1:
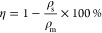
1where η *is* the porosity, ρ_s_ is the density of the scaffold [mg·mm^–3^], and ρ_m_ is the density of the scaffold material [mg·mm^–3^].

The ρ_s_ was calculated using eq 2:
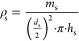
2where ρ_s_—density of scaffold [mg·mm^–3^] *m*_s_—measured dry mass of scaffold [mg], *d*_s_—measured diameter of the scaffold [mm], *h*_s_—measured height of the scaffold [mm].

ρ_m_ was calculated using eq 3:
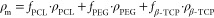
3where ρ_m_—calculated density of scaffold material [mg·mm^–3^], *f*_PCL_—volume fraction of PCL [–], ρ_PCL_—density of PCL [mg·mm^–3^], *f*_PEG_—volume fraction of PEG4 or PEG20 [–], ρ_PEG_—density of PEG4 or PEG20 [mg·mm^–3^], *f*_β-TCP_—volume fraction of β-TCP [–], ρ_β-TCP_—density of β-TCP [mg·mm^–3^].

The volume fractions were calculated from the mass fractions, as in eq 4 (β-TCP was used as an example):
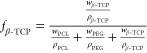
4where *f*_β-TCP_—a volume fraction for β-TCP [–], *w*_PCL_—mass fraction of PCL [–], ρ_PCL_—density of PCL [mg·mm^–3^], *w*_PEG_—mass fraction of PEG4 or PEG20 [–], ρ_PEG_—density of PEG4 or PEG20 [mg·mm^–3^], *w*_β-TCP_—mass fraction of β-TCP [–], ρ_β-TCP_—density of β-TCP [mg·mm^–3^].

### 2.6 Scanning Electron Microscopy

The morphologies of the foils and scaffolds were investigated by using scanning electron microscopy (SEM; SU8230, Hitachi). The samples of each investigated material were placed on an SEM stub using conductive carbon tape. The foils were coated with a 10 nm 80-to-20 gold–palladium (Q150T, Quorum) layer, and the scaffolds were coated with a 15 nm layer of gold (K550X Emitech, Quorum Technologies). Images were collected with a 5.0 kV accelerating voltage and 6 mm working distance using upper and lower detectors of scattered electrons (SE(UL)).

### 2.7 Fourier Transform Infrared Spectroscopy in Attenuated Total Reflectance

The presence of PEG in the scaffolds, in variants before and after PEG leaching, was investigated by using Fourier transform infrared spectroscopy (FTIR). A Nicolet 6700 spectrometer (Thermo Fisher Scientific) equipped with a SmartOrbit high-performance diamond single-bounce attenuated total reflectance (ATR) accessory was used. Spectra were analyzed using OMNIC 8.3 software (Thermo Fisher Scientific).

### 2.8 Thermogravimetric Analysis

Thermogravimetric analysis (TGA) measurements were performed by using a TGA/DSC 3+ system (Mettler Toledo). The analysis was conducted within a temperature range of 30–750 °C, at a heating rate of 2 °C·min^–1^, under a synthetic air flux of 60 mL·min^–1^ (≥99.999%, Multax) to provide an oxidizing atmosphere for the analysis. An alumina sample holder was used for the measurements.

### 2.9 Micro-CT Scanning

The scaffolds were scanned by using a microfocused X-ray tomographic system (MICRO XCT-400, Xradia, Zeiss). For each sample, 1000 projection images were recorded with an exposure time of 4 s and a magnification of 4×. Voxel size was 4.5 × 4.5 × 4.5 μm^3^. The volume was reconstructed with instrument software (XMReconstructor) and was then exported to an Avizo Fire (FEI, Thermo Scientific) for further 3-dimensional image analysis.

### 2.10 Alizarin-Red S Staining

The availability of calcium moieties on the surface of the 3D-printed scaffold was determined by using Alizarin-Red S staining. The protocol was adapted from Wojasiński et al.^51^ First, each material (*n* = 6) was incubated with 1 mL of a 40 mM (pH 4.1) solution of Alizarin-Red S in distilled water for 1 h. Each material was then washed with distilled water three times with 5 min of shaking each time on a DTS-4 shaker. After washing, Alizarin-Red S was extracted from the materials using 1 mL of a 10% w/v solution of cetylpyridinium chloride monohydrate in distilled water. Extracts were collected and transferred to a 96-well plate for absorbance measurement (405 nm) using a Spectrostar Nano microplate spectrophotometer (BMG Labtech). Results are presented as the mean optical density (OD) ± SD (*n* = 18). Representative samples of 3D-printed scaffolds after Alizarin-Red S staining were photographed with an iPhone 13 (Apple).

### 2.11 Uniaxial Compression Testing

The scaffolds were mechanically tested under uniaxial compression by using an INSTRON 3345 (INSTRON) universal testing machine with a 5 kN load cell. The cylindrical specimens (*n* = 5) were tested at a deformation rate of 1 mm·min^–1^. Tests were conducted until the sample deformation reached 0.6 mm·min^–1^ (60%). The test procedure was performed according to the ASTM D695 standard. The compression modulus was calculated from the initial slope of the stress–strain curve before the plateau region, as shown in eq 5:
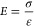
5where *E* is the compression modulus [MPa], σ is the compression stress [MPa], and ε is the compressive strain [–].

### 2.12 Cell Culture

The following cell lines were selected for this study: L929 cells (mouse fibroblasts) and MG63 cells (human osteosarcoma). Both cell lines were cultured in DMEM supplemented with 10% (v/v) FBS, 100 U·mL^–1^ penicillin, and 100 μg·mL^–1^ streptomycin, further referred to as supplemented DMEM, in 75 cm^2^ cell culture flasks and maintained at 37 °C in an incubator with 5% CO_2_. The culture was monitored under a microscope every 2 days, dissociated, and divided when the cells were near full confluency. The cell dissociation protocol was based on trypsin-EDTA solution. The cell concentration was measured in a Thoma cell counting chamber (Marienfeld).

### 2.13 Extract Cytotoxicity

Following the PEG leaching process, 3D-printed scaffolds (*n* = 2 for each variant) were sterilized by immersing them in a 70% ethanol solution and then dried in a laminar flow hood for 30 min. Next, the scaffolds were immobilized using polypropylene inserts in a 24-well plate and incubated in 1.5 mL of supplemented DMEM for 24 h to produce scaffold extracts. Additionally, a 0.1% Triton X-100 solution in supplemented DMEM was prepared and incubated as a positive control (*n* = 2) during the same period. Supplemented DMEM with a polypropylene insert served as a negative control and was stored in an incubator for 24 h (*n* = 2). The L929 cell line was cultured in 96-well plates at a concentration of 10^5^ cells·mL^–1^ in 100 μL of the culture medium per well. After 24 h, the DMEM was replaced with extracts and control samples (*n* = 6 for each sample). Following 24 h of cultivation with extracts and control samples, the cells were rinsed twice with 100 μL of DPBS. Next, 100 μL of DMEM without phenol red and supplementation was added to each culture well. Then, 70 μL of XTT solution with an electron-coupling reagent was added to each culture well, and the cells were incubated for 4 h. After the XTT was reduced to formazan pigment by viable cells, 100 μL of assay medium from each well was transferred to a new 96-well plate and the absorbance was measured at 475 nm using a Spectrostar Nano microplate spectrophotometer.

### 2.14 Cell Proliferation

Each variant of 3D-printed scaffolds after the PEG leaching procedure (*n* = 2 per culture period) was sterilized by immersion in 70% ethanol solution and dried in a laminar flow hood for 30 min. The dried scaffolds were immobilized by using polypropylene inserts in a 24-well plate. To each well, 1 mL of MG63 cell suspension (2 × 10^4^ cells·mL^–1^) in supplemented DMEM was added and incubated for 1, 3, and 7 days at 37 °C in an incubator with 5% CO_2_, with exchanging culture medium every 48 h. After each incubation period, the culture medium was removed from the wells, and samples were washed twice with a DPBS solution to wash off the culture medium. Next, the samples were treated with 1 mL of a 4% (w/v) DPBS solution of PFA for 15 min, 1 mL of 0.2% (v/v) DPBS solution of Triton X-100 for 5 min, 1 mL of 0.1% (m/v) DPBS solution of BSA for 1 h, 500 μL of 2.5% DPBS solution of Alexa Fluor 488 Phalloidin for 15 min, and 500 μL of 300 nM DAPI solution in DPBS for 5 min. After each step, the samples were washed twice with 1 mL of DPBS. The prepared samples were then transferred to a microscope slide for imaging using an LSM 880 confocal laser scanning microscope (CLSM, Zeiss). The obtained images were processed using ImageJ software.^53^

### 2.15 ALP Activity and Protein Content

The osteogenic potential of the scaffolds was evaluated by using alkaline phosphatase (ALP) activity measurements. Scaffolds (*n* = 4) were prepared and MG63 cells were cultured on them for 1, 3, and 7 days in the same way as described in Section 2.14. After each period, the scaffolds were washed three times with DPBS. Each sample was then immersed in 700 μL of cold RIPA buffer and incubated at room temperature on a laboratory shaker for 10 min. A total of 400 μL of lysate from each scaffold was transferred to a new 24-well plate, mixed with 200 μL of substrate buffer (10 mM *p*-nitrophenol phosphate, 5 mM MgCl_2_, and 100 mM diethanolamine), and incubated at 37 °C. After 2 h, the reaction was terminated by the addition of 100 μL of 10% NaOH. The p-nitrophenol concentration was measured using a Spectrostar Nano microplate spectrophotometer at λ = 405 nm. The results were normalized to the total protein concentration of the lysate, which was measured by using a QuantiPro BCA assay kit. The BCA assay was performed according to the kit instructions in 96-well plates, and the samples were incubated for 2 h at 37 °C. The absorbance of the lysate was measured using a Spectrostar Nano microplate spectrophotometer at λ = 562 nm.

### 2.16 Statistical Analysis

The significance of differences among the mean values of the measured properties of samples was verified through one-way ANOVA using Tukey’s posthoc test in OriginPro 8 (OriginLab Corporation) software. Results with *p* < 0.05 were considered statistically significantly different.

## 3 Results

### 3.1 Physicochemical Properties

#### 3.1.1 PCL-PEG Foils

**Figure 1:**
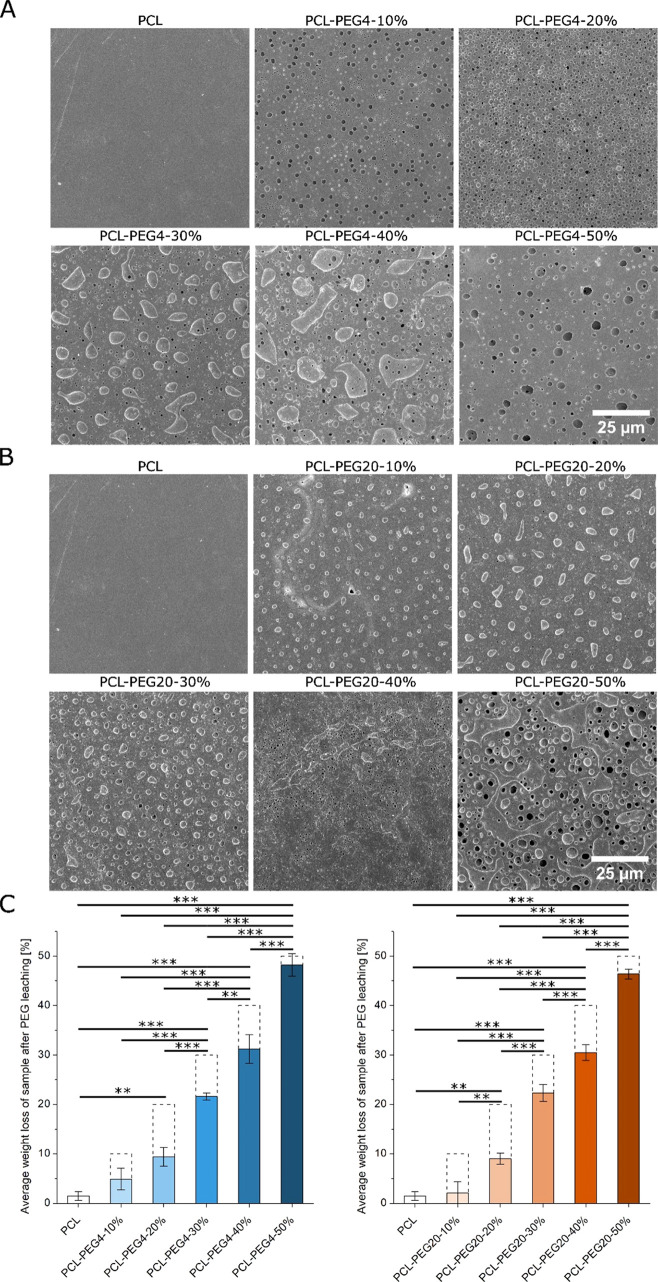
(A) SEM images of the surface of PCL-based foils with different amounts of PEG4 after the PEG leaching procedure. (B) SEM images of the surface of PCL-based foils with different amounts of PEG20 after the PEG leaching procedure. (C) Comparison of measured weight loss of PCL-based foils with different amounts of PEG4 or PEG20 after the PEG leaching procedure. Columns with dashed borders represent the weight loss percentage for theoretical 100% leaching procedure efficiency. For all variants, *n* = 4. Asterisks denote a significant difference between samples with (**) *p* < 0.01 and (***) *p* < 0.0001.

First, we produced testing samples of PCL foil and PCL foils containing 10, 20, 30, 40, and 50% (w/w) PEG4 or PEG20 (Figure S1). We also attempted to obtain foils with a 60% (w/w) PEG content, but such materials were too brittle, and they easily fragmented upon detaching from the glass plate. SEM observations of the produced foils after the PEG leaching procedure indicated that even a 10% (w/w) addition of PEG4 and PEG20 promotes the formation of 1 μm size pores on the surface of the foil (Figure 1A,B). The effect of PEG leaching on weight loss was also investigated (Figure 1C). For materials containing 10% and 20% PEG4 or PEG20, the weight loss was less than half of the weight of the PEG contained in the material. The highest level of leaching of PEG4 and PEG20 was observed in materials containing 50% PEG4 and PEG20, indicating the formation of highly connected pores. Based on observations of the possibility of creating pores in PCL-PEG materials and almost complete PEG leaching in foils containing 50% (w/w) PEG. Additionally, the change in foils’ transparency is strongly visible for 40 and 50% of PCL-PEG variants, which turned opaque white after leaching (Figure S2). Based on the SEM images, mass loss, and visual analysis, we manufactured filaments containing 50% (w/w) of PEG4 or PEG20 to PCL.

#### 3.1.2 3D-Printed Scaffold Properties

**Figure 2:**
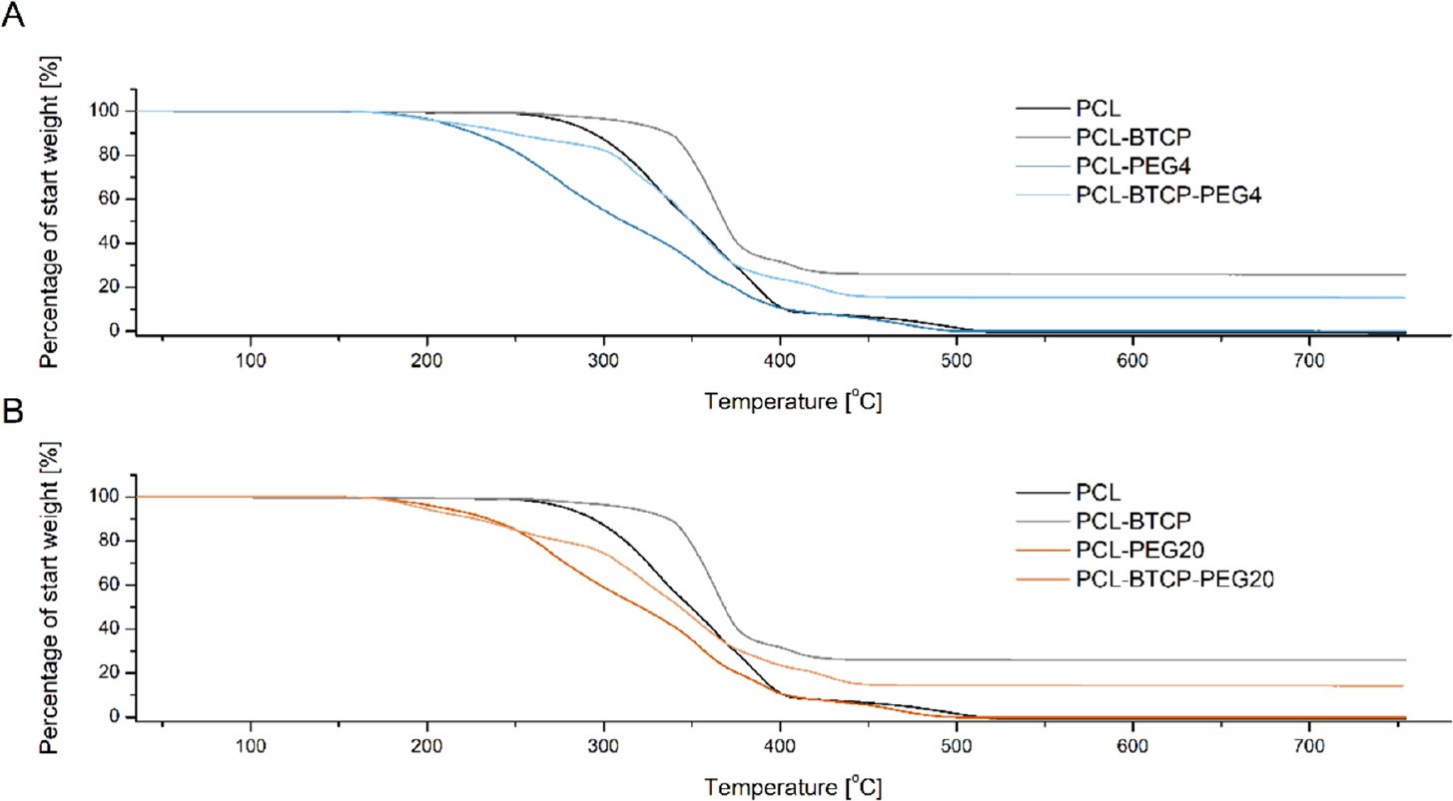
(A) TGA analysis showing the decomposition temperature and mass loss of the PCL, PCL-BTCP, PCL-PEG4, and PCL-BTCP-PEG4 materials. (B) TGA analysis showing the decomposition temperature and mass loss of the PCL, PCL-BTCP, PCL-PEG20, and PCL-BTCP-PEG20 materials.

For filament production, we employed the pressure filament extruder.^44^ Each portion of the material was sufficient to obtain a few 1 m long filaments with a diameter of 2.85 mm. TGA analysis of obtained filaments (Figure 2) shows that materials containing PEG4 (Figure 2A) and PEG20 (Figure 2B) start to degrade after crossing 175 °C, and samples without PEG4 and PEG20 start to degrade after 250 °C. In all cases, the degradation rate was slower for materials containing β-TCP. After crossing the 500 °C weight of PCL, PCL-PEG4, and PCL-PEG20 samples were near 0% of start weight, PCL-BTCP was near 25.6% of start weight, PCL-PEG4-BTCP was near 15.2% of start weight, and PCL-BTCP-PEG20 was near 14.2% of start weight. The temperatures of the melting point for PEG4 and PEG20 are slightly different −64 °C for PEG4 and 68 °C for PEG20 (Figure S3).

**Figure 3:**
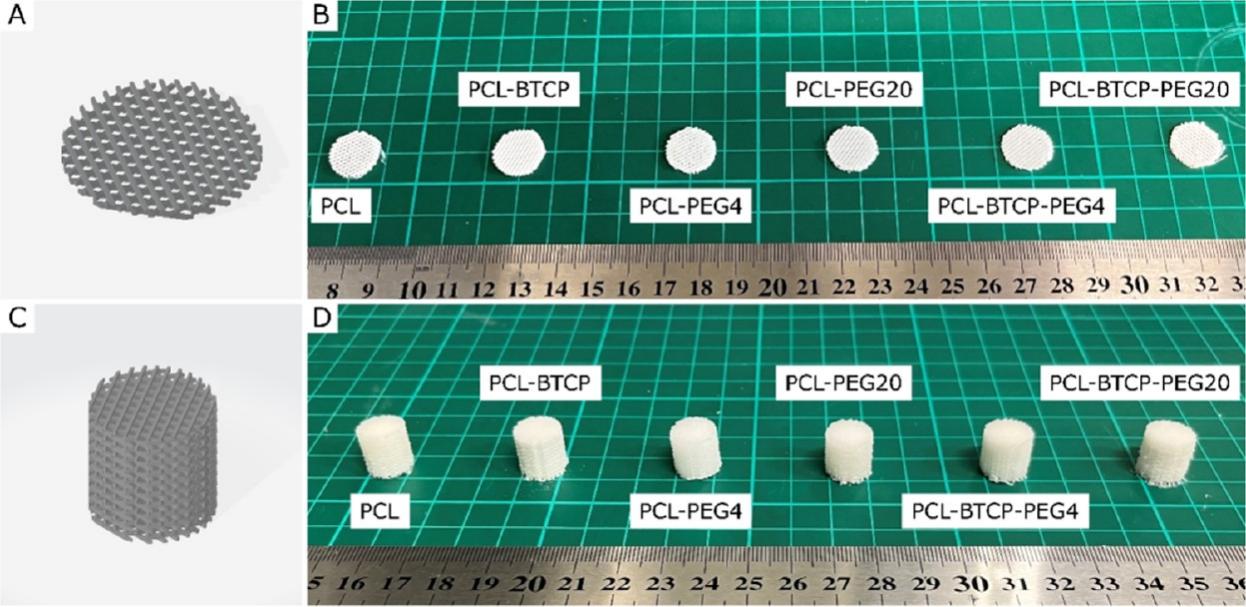
(A) 3D rendering of the scaffold for in vitro tests. (B) 3D-printed scaffolds for in vitro tests, SEM investigations, and FTIR analysis. (C) 3D rendering of the scaffold for mechanical tests. (D) 3D-printed scaffolds for mechanical compression tests.

All produced filaments (Figure S4) were suitable for 3D printing, and the PCL, PCL-BTCP, PCL-PEG, and PCL-BTCP-PEG variants of scaffolds obtained in the process were presented in Figure 3B,D. The same parameters, such as the temperature and filament feed rate, were used for the 3D printing of all scaffold variants. To check if the type of material used affects the geometry of scaffolds due to potential shrinkage/warpage, we measured the weight, height, and diameter of scaffolds and presented the results in Table 3.

**Table 3:** Mass, Height, and Diameter of 3D-Printed Scaffolds (*N* = 15)*

sample name	measured mass ± SD (*n* = 15) [mg]	measured height ± SD (*n* = 15) [mm]	measured diameter ± SD (*n* = 15) [mm]
PCL	138.1 ± 20.0^a^	1.27 ± 0.10^f^	14.91 ± 0.09
PCL-BTCP	158.7 ± 19.8^abcde^	1.23 ± 0.07	15.00 ± 0.07^i^
PCL-PEG4	128.1 ± 4.2^b^	1.18 ± 0.06^fg^	14.93 ± 0.08
PCL-PEG20	135.1 ± 17.5^c^	1.24 ± 0.10	14.96 ± 0.11
PCL-BTCP-PEG4	137.3 ± 17.9^d^	1.32 ± 0.09^gh^	14.95 ± 0.11
PCL-BTCP-PEG20	122.3 ± 9.7^e^	1.20 ± 0.04^h^	14.85 ± 0.20^i^

* Letters in superscripts indicate pairs for which result differences are statistically significant (*p* < 0.05).

**Figure 4:**
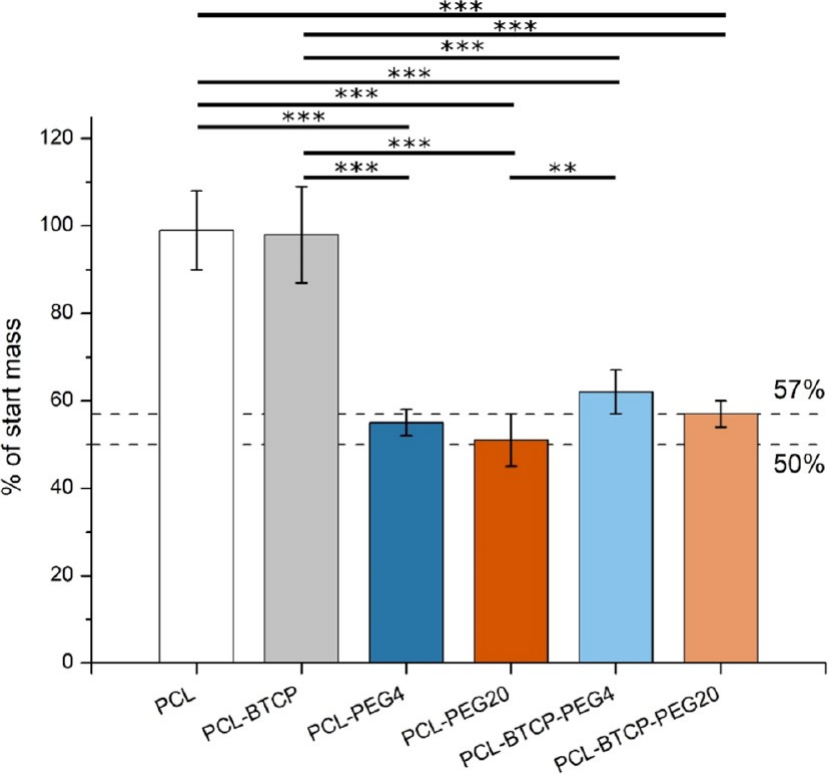
Scaffold mass after PEG leaching procedure as a % of mass before PEG leaching. The dashed lines represent the percentage of scaffold mass for theoretical 100% leaching procedure efficiency: 50% for scaffolds without β-TCP (removing all PEG mass from scaffold, 50% of PCL-PEG4 and PCL-PEG20 scaffolds mass) and 57% for scaffolds with β-TCP (removing all PEG mass from scaffold, 43% of PCL-BTCP-PEG4 and PCL-BTCP-PEG20 scaffolds mass). For all variants, *n* = 12. Asterisks denote a significant difference between samples with (**) *p* < 0.01, (***) *p* < 0.0001.

After the PEG leaching procedure, the remaining weight of scaffolds 3D-printed from the above-described filaments was near 100% of the start mass for PCL and PCL-BTCP scaffolds, 55% for PCL-PEG4, 51% for PEG20, 62% for PCL-BTCP-PEG4 and 57% for PCL-BTCP-PEG20 (Figure 4).

**Figure 5:**
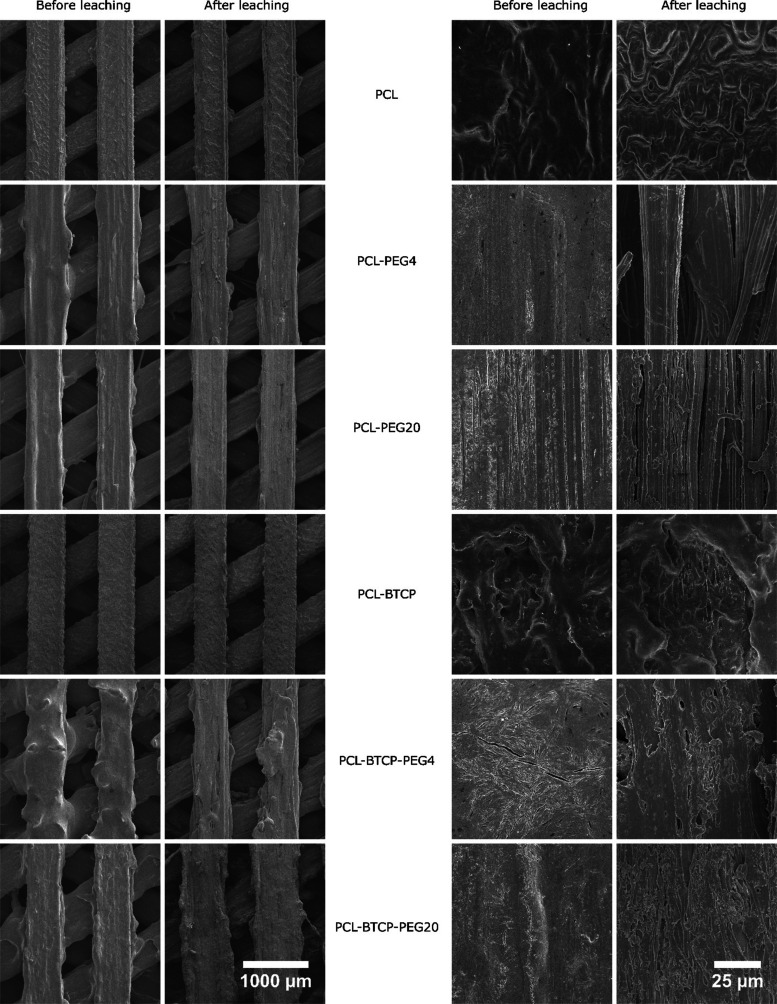
SEM images of the 3D-printed PCL, PCL-BTCP, PCL-PEG4, PCL-BTCP-PEG4, PCL-PEG20, and PCL-BTCP-PEG20 scaffolds before and after leaching. On the left, 35× magnification; at the right, 1000× magnification.

The PEG leaching procedure also influenced the surface structures of scaffolds containing PEG4 and PEG20 and the presence of characteristic chemical groups. The obtained scaffolds were examined with SEM. Images were taken at 600× magnification to show the diameter of one beam, the space between beams, and the detail of the scaffold surface. Measured diameters of beams and gaps, together with calculated porosity before and after PEG leaching, are presented in Table 4. The visible surface roughness increases in materials containing β-TCP, such as PCL-BTPC, PCL-BTCP-PEG4, and PCL-BTCP-PEG20 (Figure 5). The PEG leaching procedure had no observable influence on the surface of PCL and PCL-BTCP scaffolds, as expected, but PCL-PEG4 scaffolds and PCL-BTCP-PEG4 scaffolds were affected. The long, narrow pores and the delamination of polymer sheets became visible. A similar situation was visible in PCL-PEG20 and PCL-BTCP-PEG20 scaffolds, but the number of pores was higher, and delamination was not observed. Moreover, PCL-BTCP-PEG4 and PCL-BTCP-PEG20 have more visible particles of β-TCP than scaffolds before leaching or PCL-BTCP scaffolds without any PEG.

**Table 4:** Beam Size, Gap Size, and Porosity Value of 3D-Printed Scaffolds

sample name	measured beam size ± SD (*n* = 12) [μm]	measured gap size ± SD (*n* = 8) [μm]	porosity before leaching [%]	porosity after leaching [%]
PCL	516 ± 43	523 ± 45	45.7 ± 7.9	46.2 ± 7.8
PCL-BTCP	546 ± 38	498 ± 15	45.7 ± 6.8	46.7 ± 6.6
PCL-PEG4	577 ± 50	450 ± 33	47.7 ± 1.7	70.5 ± 1.0
PCL-PEG20	573 ± 24	505 ± 31	47.1 ± 6.8	72.4 ± 3.6
PCL-BTCP-PEG4	552 ± 59	515 ± 48	52.4 ± 6.2	72.7 ± 3.6
PCL-BTCP-PEG20	592 ± 66	464 ± 56	53.1 ± 3.7	75.3 ± 2.0

**Figure 6:**
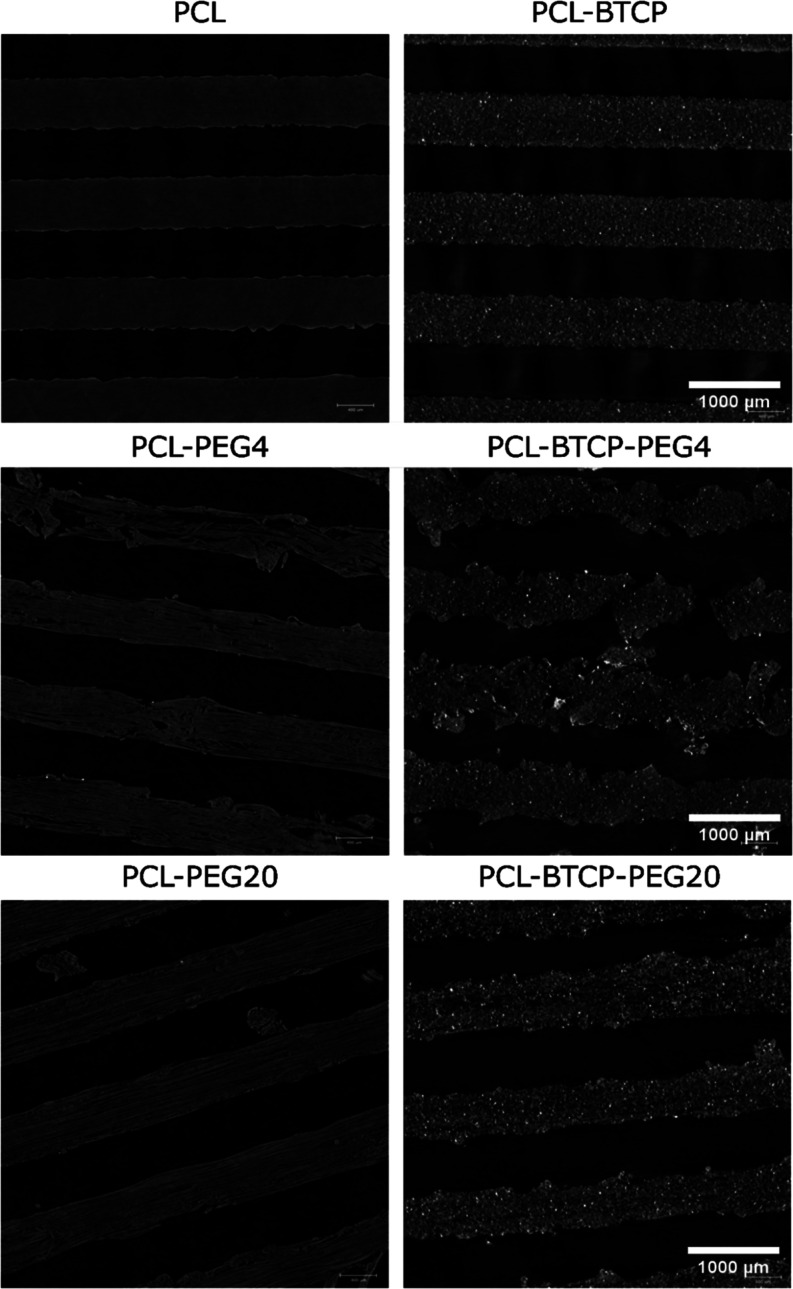
Micro-CT cross sections of 3D-printed PCL, PCL-BTCP, PCL-PEG4, PCL-BTCP-PEG4, PCL-PEG20, and PCL-BTCP-PEG20 after leaching.

Micro-CT scans also provided similar data (Figure 6). First of all, it can be seen that the PCL and PCL-BTCP materials preserve a solid structure of the beam after the PEG leaching procedure. The bright dots of calcium phosphate particles in the PCL-BTCP scaffolds are also visible. In the case of PCL-PEG4 and PCL-PEG20, a microporous structure can be observed, which results from the PEG’s leaching. However, PCL-PEG20 has ordered continuous polymer fibrous structures, while PCL-PEG4 has these fibrous structures arranged more chaotically, with visible intervals. In the case of PCL-BTCP-PEG4 and PCL-BTCP-PEG20, we notice that despite the washing procedure, the materials maintain a high content of calcium phosphate particles. The porous structure created by the PEG leaching is also visible, but PCL-BTCP-PEG4 has a noticeable chaotic structure with visible defects, while PCL-BTCP-PEG20 is arranged and without defects.

**Figure 7:**
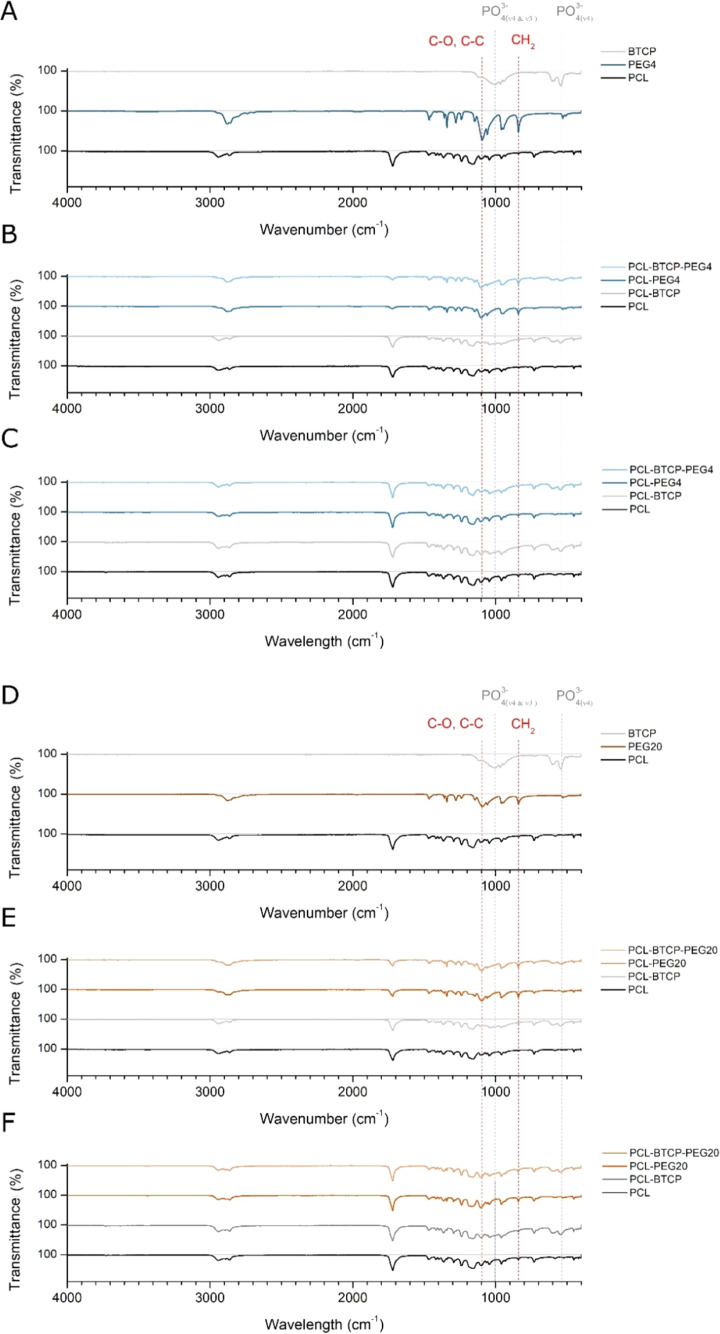
(A) FTIR-ATR spectra of pure PCL, BTCP, and PEG4. (B) FTIR-ATR spectra of PCL, PCL-BTCP, PCL-PEG4, and PCL-BTCP-PEG4 scaffolds before PEG leaching. (C) FTIR-ATR spectra of PCL, PCL-BTCP, PCL-PEG4, and PCL-BTCP-PEG4 scaffolds after PEG leaching. (D) FTIR-ATR spectra of pure PCL, BTCP, and PEG20. (E) FTIR-ATR spectra of PCL, PCL-BTCP, PCL-PEG20, and PCL-BTCP-PEG20 scaffolds before PEG leaching. (F) FTIR-ATR spectra of PCL, PCL-BTCP, PCL-PEG20, and PCL-BTCP-PEG20 scaffolds after PEG leaching.

The FTIR-ATR analysis confirms that the PEG leaching procedure removes PEG from the 3D-printed scaffolds (Figure 7). Generally, the characteristic peaks of pure PEG appear near wavenumbers 800, 1100, and 1250 cm^–1^. In the case of PCL-PEG4, PCL-PEG20, PCL-BTCP-PEG4, and PCL-BTCP-PEG20, peaks can be observed before leaching and most disappear after the leaching procedure. In addition, the peaks characteristic for pure PCL (wavenumbers 750, 1200, 1750 cm^–1^) are lower in PEG-containing materials. They are on the same level as those of pure PCL after the leaching procedure. The peaks characteristic for β-TCP (wavenumbers 550, 1000 cm^–1^) are also slightly more visible in PCL-BTCP-PEG4 and PCL-BTCP-PEG20 after the leaching procedure. As expected, the PEG leaching procedure has no observable influence on the FTIR-ATR spectra of PCL and PCL-BTCP scaffolds.

#### 3.1.3 Calcium Availability—Alizarin-Red S Staining

**Figure 8:**
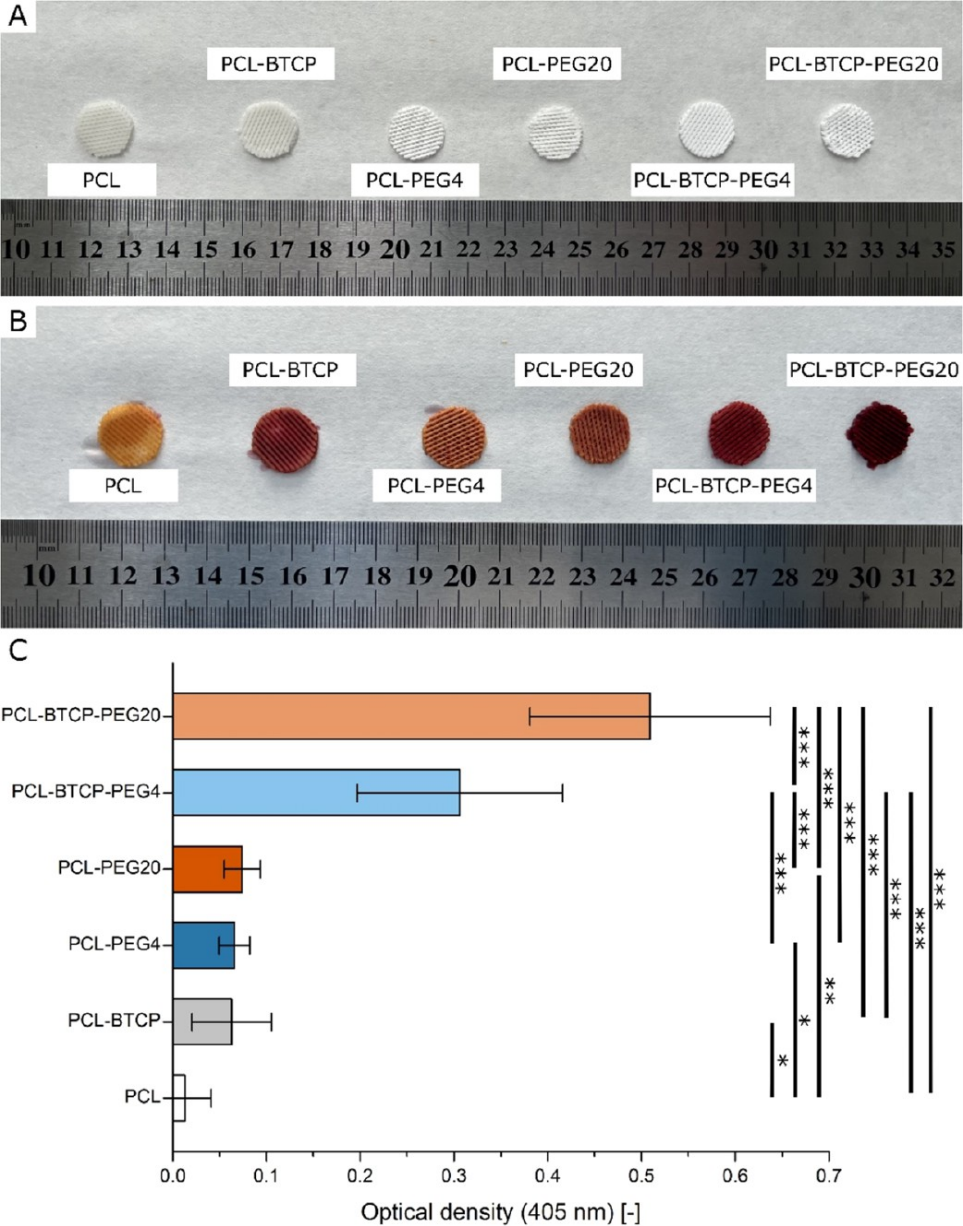
(A) 3D-printed scaffolds before staining with Alizarin-Red S solution. (B) 3D-printed scaffolds after staining with Alizarin-Red S solution. (C) Alizarin-Red S extract absorbance expressed in optical density (OD) for λ = 405 nm. For all variants, *n* = 6. Asterisks denote a significant difference between samples with (*) *p* < 0.05, (**) *p* < 0.01, and (***) *p* < 0.0001.

Scaffolds after the PEG leaching procedure were stained with Alizarin-Red S solution; it was observed that the pure PCL scaffold stained slightly yellow, and PCL-PEG4 and PCL-PEG20 scaffolds had a combination of yellow and red color. PCL-BTCP and PCL-BTCP-PEG4 scaffolds were stained red, and PCL-BTCP-PEG20 scaffolds were stained dark red (Figure 8A,B). After experimenting with the release of Alizarin-Red S into the solution of cetylpyridinium chloride monohydrate, obtained colored solutions were transferred to 96-well plates, and the absorbance was measured (λ = 405 nm; Figure 8C). Results confirmed the higher content of Alizarin-Red S in PCL-BTCP sample compared to pure PCL scaffold, what confirm detection on β-TCP. However, PCL-PEG4 and PCL-PEG20 (samples without β-TCP) showed similar results to the PCL-BTCP sample, which was likely due to the much higher porosity of the produced materials, which increased the adsorption of Alizarin-Red S despite the absence of calcium ions in tested PCL-PEG4 and PCL-PEG20 scaffolds. The biggest difference was observed for PCL-BTCP-PEG4 and PCL-BTCP-PEG20 materials compared to PCL-BTCP—absorbance read for PCL-BTCP-PEG4 scaffold was 487% higher than for PCL-BTCP, and the absorbance read for PCL-BTCP-PEG20 sample was 810% higher than for PCL-BTCP sample. In both cases, this result shows that the resulting porous structure significantly increased the availability of β-TCP particles contained in the PCL-BTCP-PEG4 and PCL-BTCP-PEG20 scaffolds.

#### 3.1.4 Compressive Strength of 3D-Printed Scaffolds

**Figure 9:**
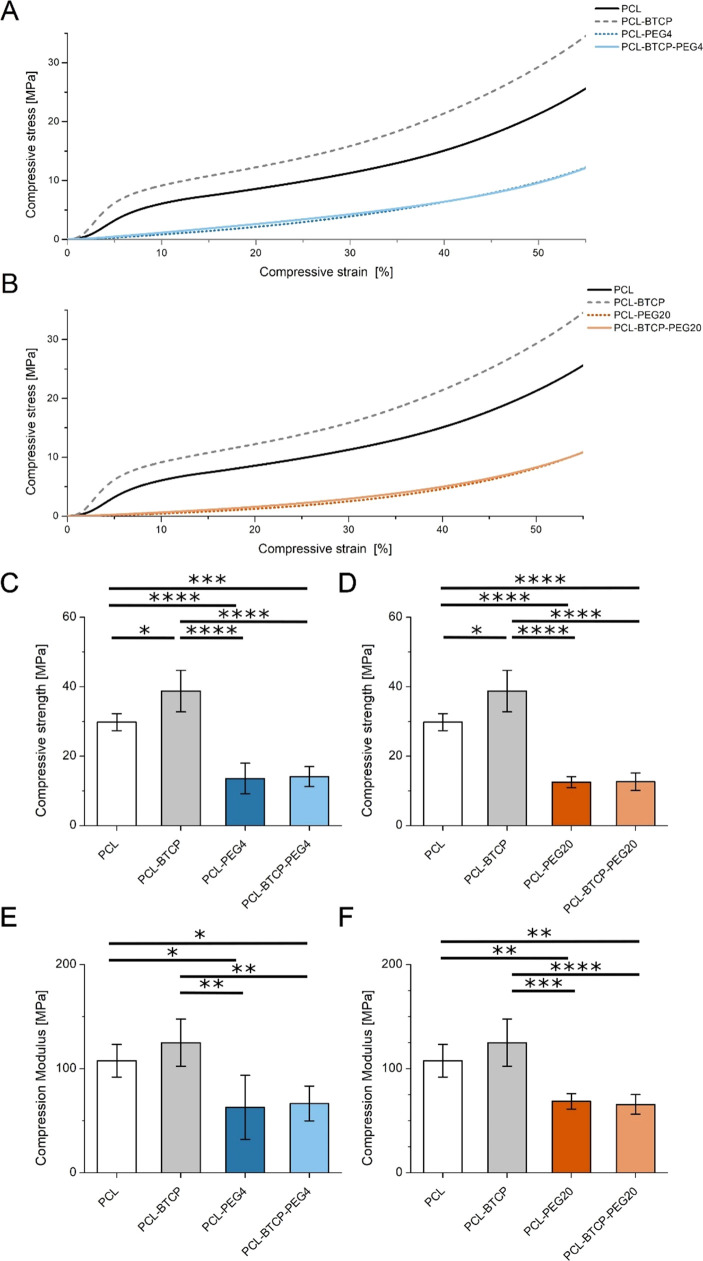
(A) Monotonic compression stress–strain representative curves for PCL, PCL-BTCP, PCL-PEG4 and PCL-BTCP-PEG4. (B) Monotonic compression stress–strain representative curves for PCL, PCL-BTCP, PCL-PEG20 and PCL-BTCP-PEG20. (C) Compressive strength from uniaxial compression test of PCL, PCL-BTCP, PCL-PEG4, and PCL-BTCP-PEG4 3D-printed scaffolds. (D) Compressive strength from uniaxial compression test of PCL, PCL-BTCP, PCL-PEG20, and PCL-BTCP-PEG20 3D-printed scaffolds. (E) Compression modulus from a uniaxial compression test of PCL, PCL-BTCP, PCL-PEG4, and PCL-BTCP-PEG4 3D-printed scaffolds. (F) Compression modulus from uniaxial compression test of PCL, PCL-BTCP, PCL-PEG20, and PCL-BTCP-PEG20 3D-printed scaffolds. For all variants, *n* = 5. Asterisks denote a sample significantly different from the others with (*) *p* < 0.05, (**) *p* < 0.01, (***) *p* < 0.001, and (****) *p* < 0.0001.

The results of mechanical tests of the obtained scaffolds are presented as graphs of the dependence of compressive stress on compressive strain (Figure 9A,B). In the case of the PCL and PCL-BTCP materials, the addition of ceramics did not affect the course of the deformation-compression curve. Adding PEG4 or PEG20 changes the behavior of scaffolds and greatly impacts the shape of the deformation-compression curve. Pure PCL scaffolds have 108 MPa of compression modulus and 30 MPa of compressive strength. For samples with the 50% addition of PEG, the value of compression modulus decreased to 63 MPa for PCL-PEG4 and 69 MPa for PCL-PEG20, with *p* < 0.0001 for both variants. The compression modulus value at maximum load was reduced to 13.6 and 12.6 MPa, respectively (Figure 9C–F). In the case of scaffolds containing β-TCP, the PCL-BTCP variant has a compression modulus value near 125 MPa and compressive stress at a maximum load near 39 MPa. The compression modulus of PCL-BTCP-PEG4 scaffolds drops to 67 MPa and PCL-BTCP-PEG20 to 66 MPa; compressive stress at a maximum load lowers to 14.1 and 12.6 MPa respectively (Figure 9C–F).

### 3.2 Bioactive Properties

#### 3.2.1 Cytotoxicity Properties of 3D-Printed Scaffolds

**Figure 10:**
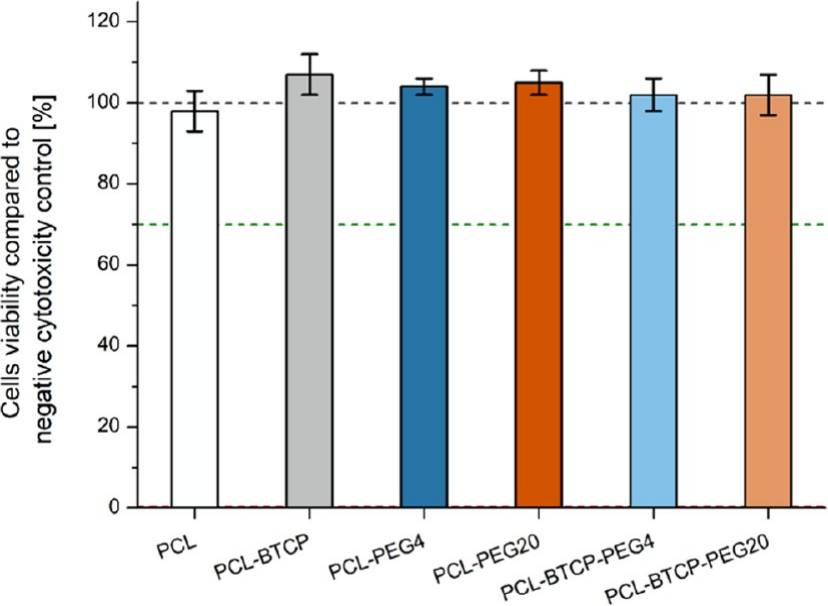
L929 cell viability after 24 h incubation with scaffolds’ liquid extracts. The black dashed line indicates the cell viability value obtained for negative cytotoxicity control. The green dashed line indicates the cytotoxicity value below which samples are considered cytotoxic. The red dashed line indicates the cell viability value obtained for positive cytotoxicity control. There was no significant difference in cell viability between tested samples and the negative cytotoxicity control or between tested samples (*p* > 0.05).

Performing a cytotoxicity test is an essential step in evaluating the manufactured materials, mainly due to detecting potential cytotoxicity resulting from organic solvents or unforeseen changes arising in the material during manufacturing. We used XTT assay to determine if scaffolds released any toxic agent that might slow down or shut down the metabolic activity of cells, resulting in a decrease in cells’ viability. The method is based on ISO EN 10993-5 standard, “Biological evaluation of medical devices”. In our research, XTT cytotoxicity tests on the L929 cell line were conducted on 24 h extracts. According to the ISO EN 10993-5 protocol’s criteria, a value of cells’ viability in contact with extract or sample should be <70% of the negative control to recognize cytotoxic properties of materials tested in vitro. Our results show a lack of cytotoxic properties for every investigated 3D-printed type of scaffold (Figure 10)—all samples exhibit over 95% cells’ viability compared to negative cytotoxicity control.

#### 3.2.2 3D-Printed Porous Scaffolds Influence on Mg63 Cells Proliferation, Protein Content, and ALP Activity

**Figure 11:**
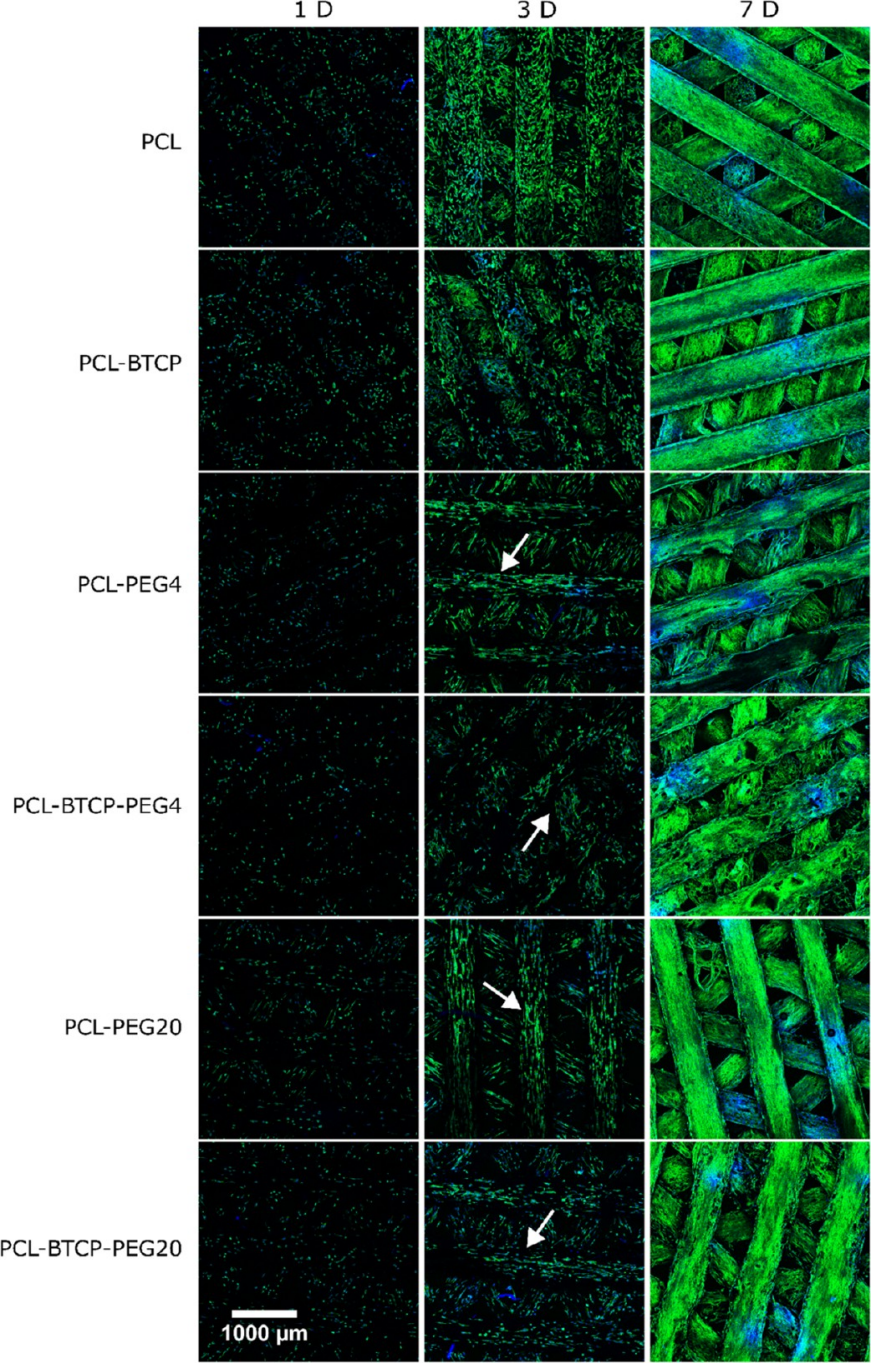
Wide view CLSM images of MG63 cell culture on surfaces of PCL, PCL-BTCP, PCL-PEG4, PCL-PEG20, PCL-BTCP-PEG4, and PCL-BTCP-PEG20 scaffolds after 1, 3, and 7 days of cultivation. White arrows indicate examples of cells aligned in the surface pore direction (parallel to beams) on materials after cultivation for 3 days of cultivation.

Proliferation studies of MG63 cells also confirmed a lack of cytotoxicity in long-time cultivation. We observed the adhesion of MG63 cells to all tested material variants (Figure 11). No visible difference in MG63 cell number on the surface of the tested scaffold occurred, depending on the type of PEG, surface porosity, and β-TCP presence in the case of composites. MG63 cells completely overgrown all scaffolds within 7 days (Figure 11). However, based on the observations, it can be assumed that stretched porous structures developed on the surface of scaffolds by PEG leaching from PCL-PEG4, PCL-PEG20, PCL-BTCP-PEG4, and PCL-BTCP-PEG20 scaffolds had a significant impact on the orientation of the cells and the direction of growth. The effect became observable, especially on 3rd day of cultivation—examples of such behavior were marked with white arrows (Figure 11).

**Figure 12:**
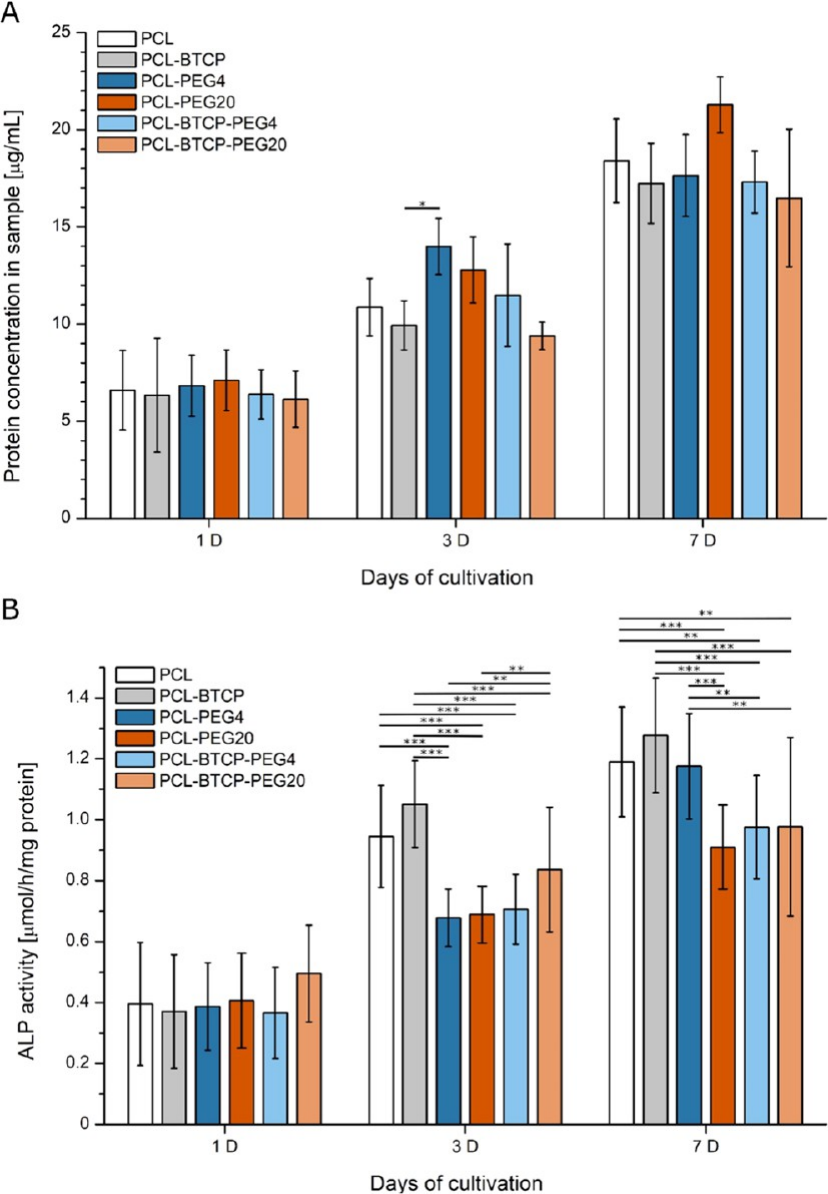
(A) Protein concentration results for PCL, PCL-BTCP, PCL-PEG4, PCL-PEG20, PCL-BTCP-PEG4 and PCL-BTCP-PEG20 scaffolds after 1, 3, and 7 days of cultivation with MG63 cells. For all variants *n* = 4. Asterisks denote a sample significantly different from the others with (*) *p* < 0.05. (B) ALP activity assay results, normalized to protein content, for PCL, PCL-BTCP, PCL-PEG4, PCL-PEG20, PCL-BTCP-PEG4, and PCL-BTCP-PEG20 scaffolds after 1, 3, and 7 days of cultivation with MG63 cells. For all variants *n* = 4. Asterisks denote a significant difference between samples with (*) *p* < 0.05, (**) *p* < 0.01, and (***) *p* < 0.001.

The BCA assay confirms cell proliferation by a steady increase in protein concentration in the samples (Figure 12A). At the same time, no significant differences were observed for the increase in the amount of protein—the only exception is a significant difference in protein content between MG63 cells cultured on PCL-BTCP and PCL-PEG4 materials for the 3rd day of cultivation. However, this difference disappears on the 7th day of culture. In the cases of the ALP activity assay, results show no significant differences for the 1st day of culture. After 3 and 7 days of culture, an increase in ALP activity (correlated to protein content) was noticed for all materials, but it was observed that almost for all scaffolds with a porous structure obtained by PEG leaching, regardless of the presence of calcium phosphate, ALP activity was significantly lower than for materials with a solid structure (Figure 12B). Results of ALP activity uncorrelated with the protein content are presented in Figure S5.

## 4 Discussion

Despite the years of research and development of new materials for bone implants, autologous bone grafts remain the most popular material to fill bone defects. Numerous artificial bone implant production technologies described in the scientific literature require specialized production facilities and qualified personnel, which are time-consuming and expensive.^15^ In our previous publication about 3D-printing polymer-ceramic scaffolds, we pointed out that producing filaments for FFF-type 3D printers can be the most difficult step in obtaining new materials.^44^ This is due to the need for a screw extruder, which has quite high material consumption, an important factor during work with expensive polymers and bioactive substances. An alternative is to print scaffolds bypassing the production of filaments, such as by using bioprinters, which allow the work on smaller volumes of materials; however, bioprinters are advanced and expensive research equipment. Our solution to this problem is a pressure filament extruder—an open-sourced, simple-to-build device with a total cost of about €350.^45^ The device allows the production of meter-long filaments of acceptable quality using only about 30 g of the tested material. The design of the pressure extruder allows for a quick change of material, which makes it possible to obtain several variants of filaments from different materials in 1 day, without cross-contamination. To demonstrate that it is possible to simply produce more advanced materials for 3D-printing bone implants, we used our device to receive filaments and 3D-printed scaffolds consisting of PCL, β-TCP, and one of the two types of PEGs.

We started our research by analyzing the effect of PEG additives on the geometry of the obtained scaffolds. The larger-than-designed width of the beams was achieved by the 3D printer being programmed to extrude more material, which helped improve the adhesion of the scaffold to the 3D-printer table and minimize appearance discontinuity defects. At the same time, it made it possible to obtain scaffolds with the width of beams and gaps within the limits of optimal bone regeneration^7−9,54^—500 μm for both. Despite differences in material composition, there was no significant difference in the diameters of the acquired scaffolds. Some statistically significant differences in heights between PCL and PCL-PEG4, PCL-PEG4 and PCL-BTCP-PEG4, and PCL-BTCP-PEG4 and PCL-BTCP-PEG20 scaffolds were observed; however, the highest difference did not exceed 11% of the height value of the lowest sample. The largest differences in weight between scaffold variants reached a maximum of 30%, but such differences are due to the presence of β-TCP, whose density is almost 3 times higher than that of PCL or PEG.

Moreover, we also used a simple method of removing PEG from the obtained materials based on washing samples in 70% EtOH. This method allows the development of surface pores without expensive equipment or dangerous reagents and can be used for simultaneous sterilization of the scaffolds. In the presented process, we fabricate scaffolds with developed micrometer-scale porous structures on the surface of the scaffold, visible in the SEM images. This effect was not achieved in 3D-printed materials based on a mixture of PLA and PEG from other research teams.^42,43^ We postulate that the main cause of the appearance of pores in the presented study is a higher concentration of PEG (up to 50% w/w) than in already published papers. Also, the temperature necessary for melting PLA is high enough to start the degradation of PEG, as we show in our TGA analysis result. This degradation can lead to insufficient surface pore formation. All melting processes presented here (filament production and 3D printing) were performed below the degradation temperature. Analyzing other research teams’ publications, we noticed that when they produced PCL materials with surface pores by removing particles of salts or sugars, the resulting pores had mostly cubic geometry and size corresponding to the size of used crystals.^27,41^ Different spherical pore geometry can be obtained by solvent evaporation, like in the case of elastic porous PCL-HAp scaffolds described by Jakus et al.^55^ In our method, pores developed on the surface of our scaffolds had a high aspect ratio and elongated shapes. PCL and PEG, according to studies of Luo et al.^56^ or Tian et al.^57^ are polymers that are immiscible and form separate crystalline domains. Therefore, in our case, even homogeneous-looking PCL-PEG materials are actually a matrix of PCL with suspended spherical PEG crystals. Therefore, when leached, characteristic circular pores are formed, visible in the SEM images in Figure 1. However, the situation changes significantly when such materials are melted and pressed through the extruder head. Then, the polymer chains, both PCL and PEG, are stretched, from chaotic to aligned and oriented parallel to the movement of the 3D printer’s nozzle and the extrusion direction. This effect is consistent with the description of the behavior of polymers during FFF-type 3D printing presented by Gantenbein et al.^58^ or Ghodbane et al.,^59^ and is both visible in the form of porous longitudinal structures on the surface of scaffolds visible in SEM images, and in the porous structure of scaffolds visible in images showing results from computed microtomography. After the PEG leaching procedure, molecules of PEG were washed out and fibrous structures of PCL with gaps between them became exposed. This effect is very noticeable in the images obtained by the micro-CT results, especially for the PCL-PEG4 and PCL-PEG20 materials. The difference between using 4 and 20 kDa PEG is also revealed during SEM and micro-CT analysis. Scaffolds containing 20 kDa PEG have an orderly structure and no visible defects, while scaffolds containing 4 kDa PEG look more knotted, and there are visible breaks in the continuity of the polymer fibrous structures. The mechanism of pore formation likely involves PEG precipitating out of solution in PCL as the fibrous structures cool. PEG20 precipitates earlier at higher temperatures, making the pores more regular.

The effect of obtaining fibrous structures by leaching water-soluble polymer was described by Kim et al., who made PCL/PVA materials for biomimetic microfibril muscle structure.^60^ The weight loss of the scaffolds after the PEG leaching procedure equals the weight of the contained PEG, proving that the presented method allows for the development of connected pores, which facilitates the penetration of 70% EtOH solution into the scaffold beams. All 3D-printed scaffolds have a porosity value about 50%, which is a common value for bone implants obtained by other research groups^6^ After the PEG leaching procedure there is also a noticeable increase in the porosity of PEG-containing materials by 20–25 percentage points with maximum porosity of about 75% in the case of PCL-BTCP-PEG20 scaffolds, which is a typical value for human femoral bone head,^61^ and is comparable to porosity obtained by other methods like salt crystals leaching.^62^ The materials we obtained, both before and after PEG leaching, have porosity within the porosity range typical for cancellous bones, which is 50–90% (in comparison, compacted bones have porosity at 10%).^63^

The main aim of this paper is to investigate how the proposed scaffold manufacturing method affects the availability of calcium contained in the scaffolds. The PEG leaching after 3D printing increases the scaffolds’ surface porosity in the proposed process. When scaffolds contain β-TCP particles, the increased surface porosity should increase the number of such particles on the surface of the scaffold. Thus, the number of calcium ions close to the scaffold surface should also increase. Alizarin-Red S staining showed the availability of those ions within the scaffolds without any cells present. In that way, we can confirm the hypothesis that increased surface porosity reveals more β-TCP particles in PCL-BTCP scaffolds and opens up the availability of calcium ions for further cell seeding. Our study showed that the staining effect of PCL and PCL-BTCP scaffolds with Alizarin-Red S was similar to the result of the study by Hung et al.^64^ or Abbasi et al.,^65^ where pure PCL turned yellow, and materials containing calcium phosphate or bone fragments turned red. An interesting observation is that PCL-PEG4 and PCL-PEG20 scaffolds after the PEG leaching procedure and staining with Alizarin-Red S were redder than those of pure PCL scaffolds. Moreover, PCL-PEG4 and PCL-PEG20 were stained similarly to PCL-BTCP scaffolds, probably due to stain entrapment within the pores. In the case of our PCL-BTCP-PEG4 and PCL-BTCP-PEG20 materials, forming a porous structure significantly increased the availability of β-TCP particles, as shown by SEM and micro-CT images and Alizarin-Red S staining. Materials with higher ceramic or bone content produced and described by other research teams^55,64,66^ were visually less red than PCL-BTCP-PEG20 scaffolds. This difference is also visible in the quantitative study—scaffolds with the calcium phosphate coatings, developed by Abbasi et al.,^65^ have 10–20 times lower optical density per gram of material than presented here PCL-BTCP-PEG4 and PCL-BTCP-PEG20 scaffolds in the Alizarin-Red S extraction solutions. This confirms the influence of porosity on the exposition of β-TCP particles on the surface of scaffolds produced by using the described method. The presented method allows for the production of materials with a lower ceramic content relative to the polymer while still maintaining the same or higher level of calcium availability as materials with a higher ceramic content.

The porosity of the material has a strong influence on the mechanical properties—in particular, an increase in porosity leads to a decrease in the values of compressive strength and compressive modulus^67^ The effect of porosity on the compression modulus of PCL materials was presented in the work of Guarino et al.;^68^ compressive strength and compressive modulus value decrease was observed by Xia et al. for their porous PCL-HA materials.^69^ However, in the case of salt-leached materials described in the literature, the stress–strain curve retains its sigmoid but flattened shape, even in variants with the highest porosity. In our case, the curve no longer has a sigmoidal shape and remains typical for elastomer materials.^70^ We think this effect results from long, hollow spaces mixed with structures made of ordered parallel PCL fibrous structures. The compression modulus of all our materials is lower than typical values for human bone −1.41 to 1.89 GPa,^71^ but still within the range of strength needed for applications in biomedical engineering, where the minimum compressive modulus value for hard tissue is over 10 MPa, and for soft tissue 0.4 MPa.^6^ The flexibility of the obtained materials has the advantage of quickly adjusting the scaffolds to wounds during surgery while maintaining bone regenerative properties, as shown by Jakus et al. on similar materials.^55^ A similar positive effect of PEG addition on the result of elasticity to materials containing up to 90% (w/w) hydroxyapatite was described by Cao et al.^33^ The performance of our materials can also be compared to the work of Zein et al., who contributed to the development of the first commercial 3D-printed PCL bone implant system—Osteopore.^72,73^ Their work showed how scaffold porosity (the result of designed beam geometry) affects the monotonic compression stress–strain curves for PCL scaffolds—scaffolds with 48% porosity deformed in the same way as those presented here, with plateau also around 5 MPa, and the scaffold with 76% porosity had a significantly reduced compression stress–strain curve. However, the 76% porosity material retained a curve course divided into an elastic phase, a plateau phase, and a compression phase. Our porous PCL-PEG4 and PCL-PEG20 scaffolds (70.5 and 72.4% of porosity, respectively) lost this polymer-specific compression stress–strain curve division.

The cytotoxicity assays on L929 fibroblast cells, based on ISO 10993-5 protocol,^74^ showed that no cytotoxic substances were released in amounts that reduced the viability of cells to the point considered as a cytotoxicity threshold in vitro. The production process–which includes drying and melting produced materials in relatively high temperatures compared to temperatures suitable for living organisms—eliminates residues of dichloromethane; we also demonstrated the same effect in our publication about the filament production technique.^44^ Additionally, the safety of our porous materials was proven by the proliferation experiment: MG63 cells covered the surface of all materials produced using the proposed process on the 7th day of cultivation. However, it is worth noting that the geometry of the obtained surface pores strongly influenced the orientation of the seeded cells, which is particularly evident in the images showing the condition of the culture after 3 days. In general, MG63 cultured on flat surfaces grows in a disordered and nondirectional way. On the other hand, bone-forming cells in natural conditions grow on parallel-running collagen I fibrils with hydroxyapatite nanocrystals.^75^ Although, the exact molecular mechanism of how geometry affects cell behavior is not fully understood.^76^ Nevertheless, providing similar geometrical cues on synthetic scaffolds could be beneficial. Thus, the observed alignment of MG63 cells according to the direction of the formed pores and polymer fibers on PCL-BTCP scaffolds shown here could deliver osteoconductive properties of the scaffold by biomimicry. Guo et al., in their study, showed that as many as 75% of hMSC cells had an angular range of 0–20° to horizontal beam of 3D-printed PLGA scaffolds. In the case of casted scaffolds, where the polymer fibrous structures or chains are distributed chaotically, only 40% of cells were oriented 0–20° to the horizontal beam.^77^ Another interesting example of the effect of parallel structures on cell behavior is the study by Holthaus et al., where they produced hydroxyapatite materials with microchannels with diameters ranging from 20 to 100 μm. Holthaus et al. also observed that more than 70% of human osteoblast cells arranged themselves relative (within the angular range of 0–15°) to channels with diameters of 20 μm, and more than 60% for channels with diameters of 40 μm.^78^ The influence of porous structures on the shape of MG63 cells was also shown by Song et al., where elongation of cultured MG63 cells on nanoporous alumina rise with pores size from 20 to 200 nm.^79^

Based on the results, it can be seen that the porous materials we obtained reduce the production of ALP in MG63 cells regardless of the presence of calcium phosphate. At the same time, the structure of tested materials practically has no significant differences in the increase of the protein content. Very similar results were reported by Lee et al., where they compared ALP-related gene expression and ALP activity of C3*H*/10T1/2 cells cultured on porous and solid hydroxyapatite, α-TCP, and β-TCP.^80^ According to Lee, the effect of lower ALP activity of porous materials results from an increase in the surface area of the materials, which results in less frequent cell-to-cell contacts, necessary for greater ALP production. Extensive studies of the effects of pore size on the proliferation and ALP production of MG63 cells are also presented by Lee et al.^81^ Results from MG63 cell cultures on polycarbonate membranes with pores with sizes ranging from 0.2 to 8 μm show that ALP activity for MG63 cells is lowest for pores with a size of 0.2 μm and highest for pores with sizes of 5 and 8 μm. The low effect of the presence of β-TCP on increasing the ALP activity in MG63 cells may be confusing, but Wilkesmann et al. show that this is a characteristic behavior of this cell line.^82^ Our scaffolds with parallel porous structures can be necessary for treating tissues where the fibers are ordered, e.g., muscle tissue^60^ or cartilage.^77^ In addition, such an ordered geometry of surface pores may positively affect the adhesion and growth of bone cells, among other cells.^77,78,83^ The possibility of designing this type of material may directly impact the tissue regeneration time and, thus, the speed of the patient’s recovery—therefore, it is important to develop new methods to produce multiscale porous materials.^6^

The presented method of manufacturing PCL-PEG and PCL-BTCP-PEG filaments allows for the fast and economical production of porous scaffolds using commercial 3D printers. Results show that these scaffolds have physical and biological properties comparable to materials produced in other methods but without the necessity of using expensive equipment such as bioplotters, screw extruders, or devices for high-pressure or supercritical fluids and without restrictions in the design of scaffold geometry presented by traditional particle leaching methods. Our previous work proved that cheap and fast prototyping of polymer-ceramic filaments for FFF-type 3D printers is possible. The proposed technique of bone implant fabrication allowed us to combine the material and methods to produce attractive scaffolds with developed surface for bone implantology, and the procedure used to obtain pores is also the commonly used sterilization technique, which further simplifies the whole process. In addition, the process can be adapted for other more complex materials. PCL-BTCP-PEG4 and PCL-BTCP-PEG20 are examples of advanced materials where the applied surface pore-forming technique allowed the significant enhancement of the availability of β-TCP without changing the content of ceramics in relation to PCL.

## 5 Conclusions

We demonstrated the production method of PCL and PCL/β-TCP scaffolds that contain the addition of PEG (4 or 20 kDa of molecular weight) as porogen. As a result of the leaching of PEG in ethanol aqueous solution, a developed porous structure is formed on the surface of the 3D-printed scaffolds. Fabricated materials are characterized by an oriented surface pore geometry, which strongly affects the position and growth of cells. Such geometry increases the exposure of the calcium phosphate contained in the materials and ensures high flexibility of 3D-printed scaffolds. Moreover, combining 3D printing and PEG leaching allows for integrating the surface and volume porosities into one composite scaffold. At the same time, we emphasize that the methods we described and used to produce these materials are inexpensive and easy to use, and the obtained filaments fit any FFF 3D printer. We estimate that the combination of the described material properties and the simplicity of producing these materials in the described process will significantly accelerate the development of new and better multiscale porous materials to build bone implants and implement such solutions on a larger scale in medicine.-

## Data Availability

The raw and processed data required to reproduce these findings are available to download from OSF https://osf.io/mjxsb/?view_only=75e41ed219fe4d1292fd6615f2369d4f.

## Supporting Information Available

Additional experimental details including photographs of produced PCL-PEG foils, photographs of fragments of PCL foil and PCL foil containing 10, 20, 30, 40, and 50% (w/w) of PEG4 and PEG20 before and after the PEG leaching procedure, and photographs of fragments of PCL, PCL-PEG4, PCL-PEG20, PCL-BTCP, PCL-BTCP-PEG4, and PCL-BTCP-PEG20 filaments (PDF)
