# Supplementary material for: Fabrication of 3D-Printed Scaffolds with Multiscale Porosity
Source: ACS Omega. 2024 Jun 28;9(27):29186–204. doi: 10.1021/acsomega.3c09035 (PMC11238315; doi:10.1021/acsomega.3c09035)
Supplement: Supplementary file 1 — ao3c09035_si_001.pdf [file ao3c09035_si_001.pdf]

Supporting information for:

Fabrication of 3D printed scaffolds with multi-scale porosity

Rafał Podgórski[1,], Michał Wojasiński[1], Artur Małolepszy[1], Jakub Jaroszewicz[2], Tomasz Ciach[1,3]*

[1] Warsaw University of Technology, Faculty of Chemical and Process Engineering, Waryńskiego 1, 00-645 Warsaw, Poland

[2] Warsaw University of Technology, Faculty of Materials Science and Engineering, Wołoska 141, 02-507 Warsaw, Poland

[3] Centre for Advanced Materials and Technologies CEZAMAT, Poleczki 19, 02-822 Warsaw, Poland

*Corresponding author. E-mail address: rafal.podgorski.dokt@pw.edu.pl

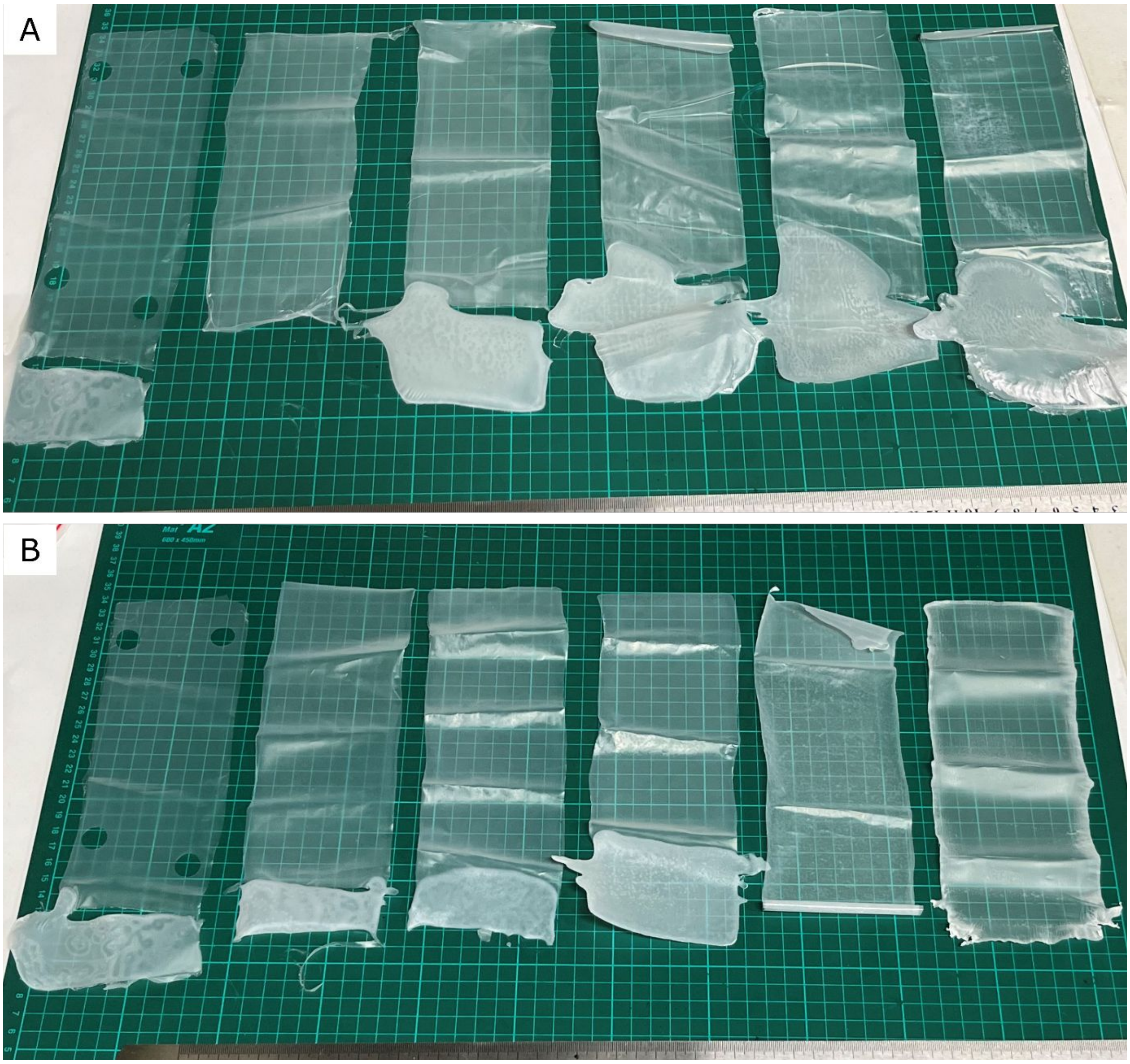

Figure S1 Images of produced PCL-PEG foils. A) From left: PCL foil and PCL foil containing 10, 20, 30, 40, and 50 % (w/w) of PEG4, B) From left PCL foil and PCL foil containing 10, 20, 30, 40, and 50 % (w/w) of PEG20

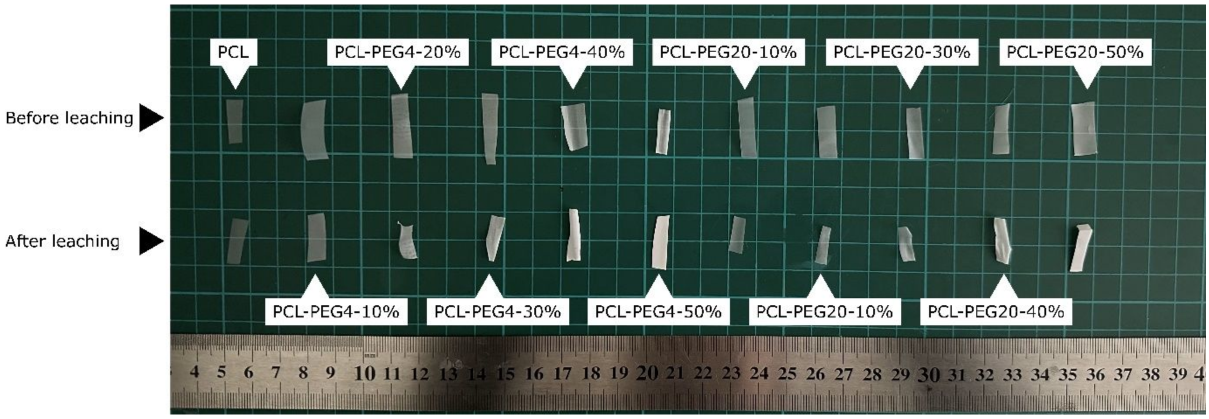

Figure S2 Fragments of PCL foil and PCL foil containing 10, 20, 30, 40, and 50 % (w/w) of PEG4 and PEG20 before and after the PEG-leaching procedure.

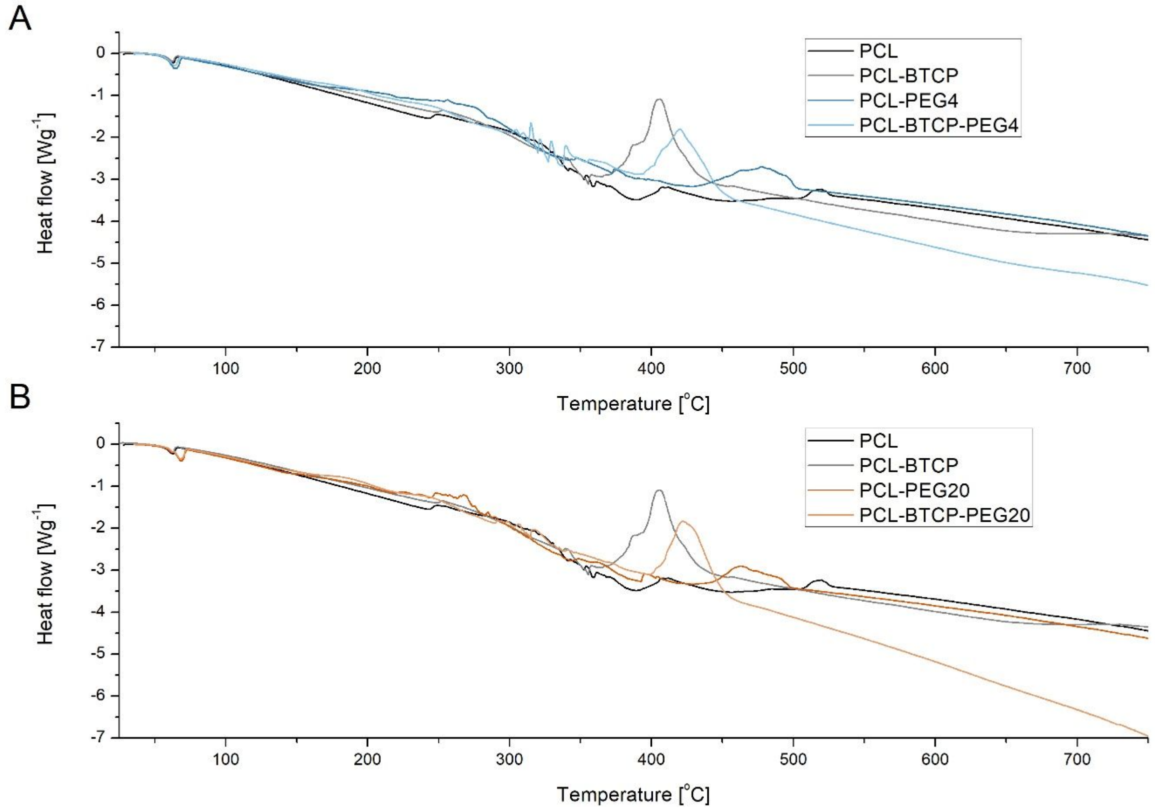

Figure S3 **A)** TGA analysis showing the heat flow under heating of the PCL, PCL-BTCP, PCL-PEG4, and PCL-BTCP-PEG4 materials. **B)** TGA analysis showing heat flow under heating of the PCL, PCL-BTCP, PCL-PEG20, and PCL-BTCP-PEG20 materials.

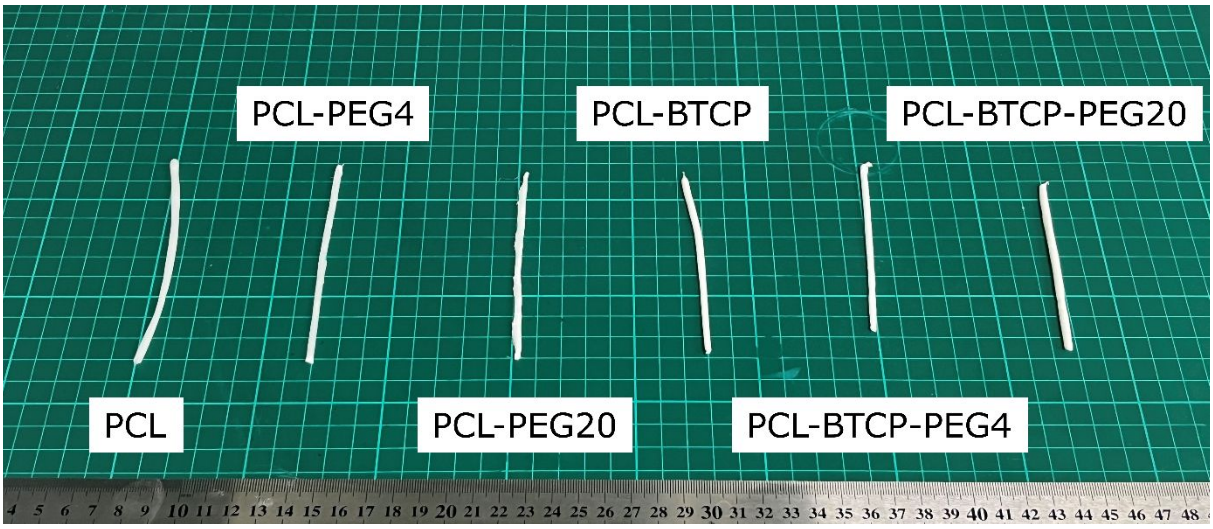

Figure S4 Filament fragments of PCL, PCL-PEG4, PCL-PEG20, PCL-BTCP, PCL-BTCP-PEG4 and PCL-BTCP-PEG20

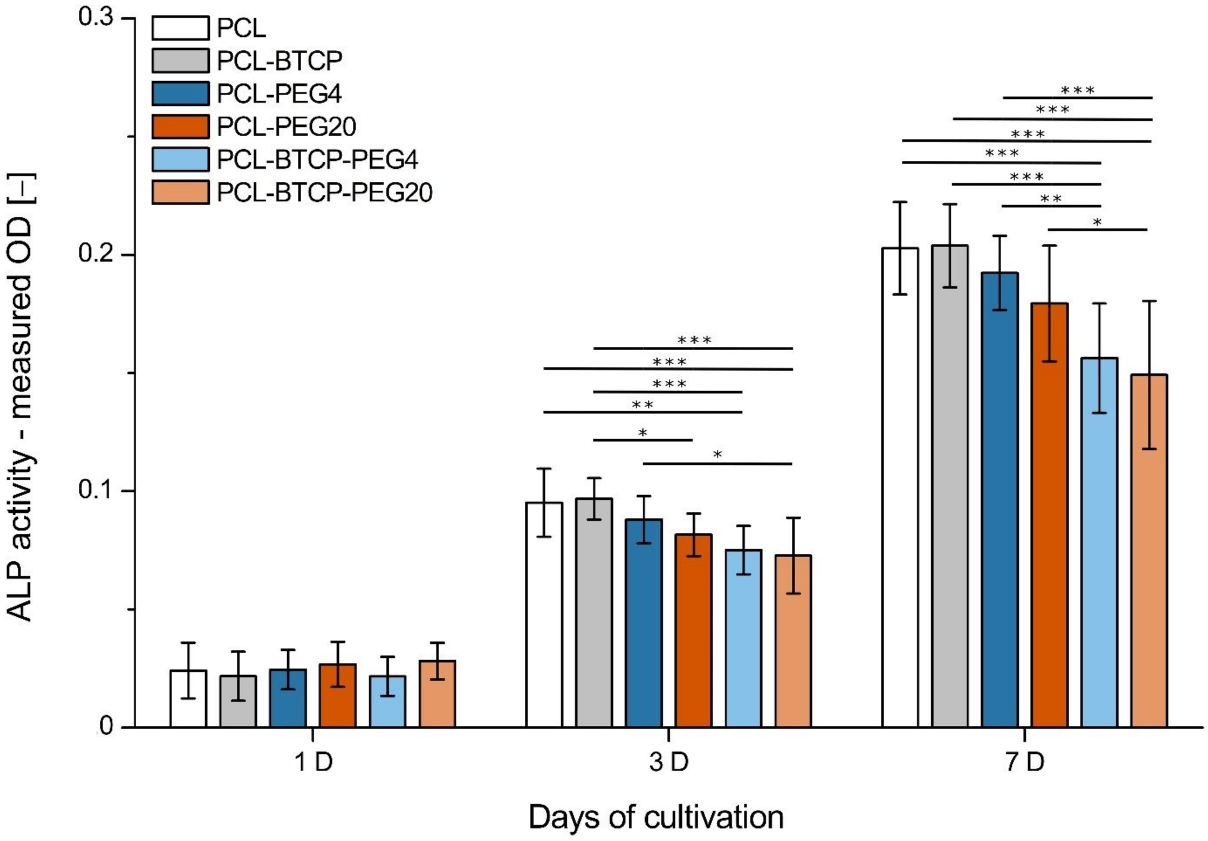

Figure S5 ALP activity assay results, presented as OD measurement of p-nitrophenol concentration for λ = 562 nm, for PCL, PCL-BTCP, PCL-PEG4, PCL-PEG20, PCL-BTCP-PEG4, and PCL-BTCP-PEG20 scaffolds after 1, 3, and 7 days of cultivation with MG63 cells.

For all variants n = 4. Asterisks denote a sample significantly different from the others with (*) $p < 0.05$, (**) $p < 0.01$, (***) $p < 0.001$
